# On the humanization of VHHs: Prospective case studies, experimental and computational characterization of structural determinants for functionality

**DOI:** 10.1002/pro.5176

**Published:** 2024-10-18

**Authors:** Monica L. Fernández‐Quintero, Enrico Guarnera, Djordje Musil, Lukas Pekar, Carolin Sellmann, Filipe Freire, Raquel L. Sousa, Sandra P. Santos, Micael C. Freitas, Tiago M. Bandeiras, Margarida M. S. Silva, Johannes R. Loeffler, Andrew B. Ward, Julia Harwardt, Stefan Zielonka, Andreas Evers

**Affiliations:** ^1^ Department of Integrative Structural and Computational Biology The Scripps Research Institute La Jolla California USA; ^2^ Antibody Discovery and Protein Engineering Merck Healthcare KGaA Darmstadt Germany; ^3^ Structural Biology and Biophysics Merck Healthcare KGaA Darmstadt Germany; ^4^ iBET, Instituto de Biologia Experimental e Tecnológica Oeiras Portugal; ^5^ Institute for Organic Chemistry and Biochemistry Technical University of Darmstadt Darmstadt Germany

**Keywords:** antibody engineering, framework residues, Hallmark, humanization, molecular dynamics, natural killer cells, NKp30, single domain antibody, VHHs

## Abstract

The humanization of camelid‐derived variable domain heavy chain antibodies (VHHs) poses challenges including immunogenicity, stability, and potential reduction of affinity. Critical to this process are complementarity‐determining regions (CDRs), Vernier and Hallmark residues, shaping the three‐dimensional fold and influencing VHH structure and function. Additionally, the presence of non‐canonical disulfide bonds further contributes to conformational stability and antigen binding. In this study, we systematically humanized two camelid‐derived VHHs targeting the natural cytotoxicity receptor NKp30. Key structural positions in Vernier and Hallmark regions were exchanged with residues from the most similar human germline sequences. The resulting variants were characterized for binding affinities, yield, and purity. Structural binding modes were elucidated through crystal structure determination and AlphaFold2 predictions, providing insights into differences in binding affinity. Comparative structural and molecular dynamics characterizations of selected variants were performed to rationalize their functional properties and elucidate the role of specific sequence motifs in antigen binding. Furthermore, systematic analyses of next‐generation sequencing (NGS) and Protein Data Bank (PDB) data was conducted, shedding light on the functional significance of Hallmark motifs and non‐canonical disulfide bonds in VHHs in general. Overall, this study provides valuable insights into the structural determinants governing the functional properties of VHHs, offering a roadmap for their rational design, humanization, and optimization for therapeutic applications.

## 1 INTRODUCTION

Heavy chain only antibodies (HcAbs), naturally occurring in cartilaginous fish (Zielonka et al., 2015) and camelids (Könning et al., 2017; Krah et al., 2016) have garnered significant interest with respect to potential biomedical applications. Within the group of HcAbs, particularly the antigen binding site of camelid‐derived HcAbs, referred to as variable domain of the heavy chain of a heavy chain only antibody (VHH), have emerged as versatile building block for the construction of mono‐ and multifunctional antibody derivatives (Arras et al., 2023; Bannas et al., 2017; Boje et al., 2024; Chanier & Chames, 2019; Jovčevska & Muyldermans, 2019; Klein et al., 2024; Lipinski, Arras, et al., 2023; Lipinski, Unmuth, et al., 2023; Pekar et al., 2020; Surowka & Klein, 2024; Yanakieva et al., 2022). In this regard, several different VHH‐based therapeutics were granted marketing access by different healthcare authorities (Duggan, 2018; Keam, 2023; Markham, 2022). Their compact size, remarkable stability, and exceptional binding specificity, coupled with ease of generation (Roth et al., 2020; Sellmann et al., 2020) renders VHHs valuable for several different applications, as reviewed elsewhere (Jin et al., 2023; Könning et al., 2017; Krah et al., 2016; Wang et al., 2022).

However, VHH domains usually must be humanized and further sequence‐optimized to be suitable for therapeutic applications (Gordon et al., 2024). VHHs often contain longer complementarity‐determining regions 3 (CDR3s) compared to conventional antibodies composed of heavy and light chain (Henry et al., 2019; Li et al., 2016; Murakami et al., 2022; Tu et al., 2020), yet they target epitopes of comparable size (Gordon et al., 2023). Sequence and structural analysis has revealed that Hallmark residues within framework region 2 (FR2; positions 42, 49, 50, 52 according to IMGT nomenclature, https://www.imgt.org/) encoded in dedicated V germline genes play a crucial role in VHH stability, diversity, and antigen binding (Nguyen et al., 2000; Nguyen & Desmyter, 2001; Soler et al., 2021; Vincke et al., 2009). These residues, which are more diverse than in canonical antibodies (Arras et al., 2023; Deszyński et al., 2022), reshape the classical light chain interface and contribute to VHH solubility and stability of the bioactive CDR3 conformation by intramolecular interactions (Kuroda & Tsumoto, 2023). Furthermore, Hallmark residues often mediate interactions with antigens (Gordon et al., 2023; Mitchell & Colwell, 2018).

Few studies have explored systematic humanization of Hallmark residues (Ben Abderrazek et al., 2011; Soler et al., 2021; Vincke et al., 2009). Vincke et al. demonstrated that humanization of two camelid‐derived VHH‐specific residues outside FR2 were neutral to VHH properties (Vincke et al., 2009). Humanization of Hallmark residues 49 and 50 (FERG/A to F*GL*G/A) resulted in higher stability, but probably lower solubility, whereas substitutions in positions 42 (F→V) and 52 (G/A→W) were detrimental for affinity due to a repositioning of CDR3. Based on these data, the authors proposed the general strategy to humanize FRs 1, 3 and 4. Within FR2, the humanization of Hallmark residues 42 and 52 is discouraged due to their demonstrated involvement in stability and antigen binding, while humanization of residues 49 and 50 is considered feasible in terms of binding affinity but might be detrimental in terms of solubility. More recently, Sulea proposed engrafting VHH CDRs into human frameworks, followed by strategic back‐mutations not only in Hallmark positions 42 and 52, but also in further key structural (Vernier) positions to balance humanness with antigen binding and stability (Sulea, 2022).

Another notable structural feature of VHHs is the prevalence of non‐canonical cysteine pairings, manifesting as either a CDR3 intraloop disulfide bond or even more often an interloop cysteine, connecting CDR3 with another residue of the VHH scaffold (Conrath et al., 2003; Govaert et al., 2012; Li et al., 2016; Mendoza et al., 2020; Muyldermans et al., 1994; Nguyen et al., 2000). Our recent analysis of the llama wild type VHH repertoire revealed that over 25% of VHH sequences carry non‐canonical disulfide bonds between cysteines within CDR3 and other cysteine residues positioned variably within the sequence (Arras et al., 2023). Intriguingly, non‐canonical cysteine residues are also present in the germline V‐genes of VHHs. This observation suggests that the somatic introduction of a cysteine at various positions within CDR3, forming a disulfide bond with germline‐encoded cysteine residues, serves as a distinct mechanism in camelids for conformational preselection and stabilization. By facilitating the generation of a wide diversity of paratope geometries, this mechanism obviously contributes to the expanded antigen‐binding repertoire of VHHs.

In this study, we present a comprehensive investigation involving the systematic humanization, experimental validation, and computational analysis of two llama‐derived VHHs, previously identified for their binding to NKp30 and their potential for natural killer (NK) cell redirection (Boje et al., 2024; Klausz et al., 2022). These VHHs, designated as VHH1 and VHH2 in accordance with our prior research, exhibit atypical Hallmark signatures (VEHG and FARS), with VHH1 additionally featuring a non‐canonical disulfide bond.

Our study focused on systematically humanizing key structural positions, including Vernier and Hallmark motifs, within the wild‐type llama VHH sequence. Subsequently, we evaluated the binding affinities, yield, and purity of the humanized variants. During this process, we successfully determined the co‐crystal structure of VHH2 bound to NKp30 and obtained the apo structure of VHH1. These structural data were complemented with AlphaFold2‐generated complex structures (Jumper et al., 2021) and molecular dynamics (MD) simulations. Together, these analyses provided valuable insights into the structural roles of Hallmark residues and the non‐canonical disulfide bond in both VHHs.

Interestingly, our findings suggest distinct mechanisms underlying antigen binding and conformational stabilization in VHH1 and VHH2, determined by specific residues. While Hallmark residues in VHH2 were found not to directly interact with the antigen, a single Hallmark residue was essential for stabilizing the bioactive conformation of CDR3. In contrast, molecular modeling studies suggest that Hallmark residues in VHH1 are directly involved in antigen interactions, with conformational stabilization of its CDR3 facilitated not by Hallmark residues, but by a non‐canonical disulfide bond.

To further elucidate the broader significance of Hallmark residues and non‐canonical disulfide bonds in CDR3 stabilization and antigen binding in VHHs in general, we conducted a thorough data‐mining analysis of diverse camelid next‐generation sequencing (NGS) repertoires and Protein Data Bank (PDB) structures. In summary, our study provides a comprehensive understanding of the structural determinants such as Hallmark residues and non‐canonical disulfide bonds in governing the functional properties of VHHs. To conclude, we provide a roadmap for VHH humanization and sequence optimization.

## 2 RESULTS

### 2.1 Sequence assessment, humanization, and optimization of VHH1 and VHH2

**FIGURE 1:**
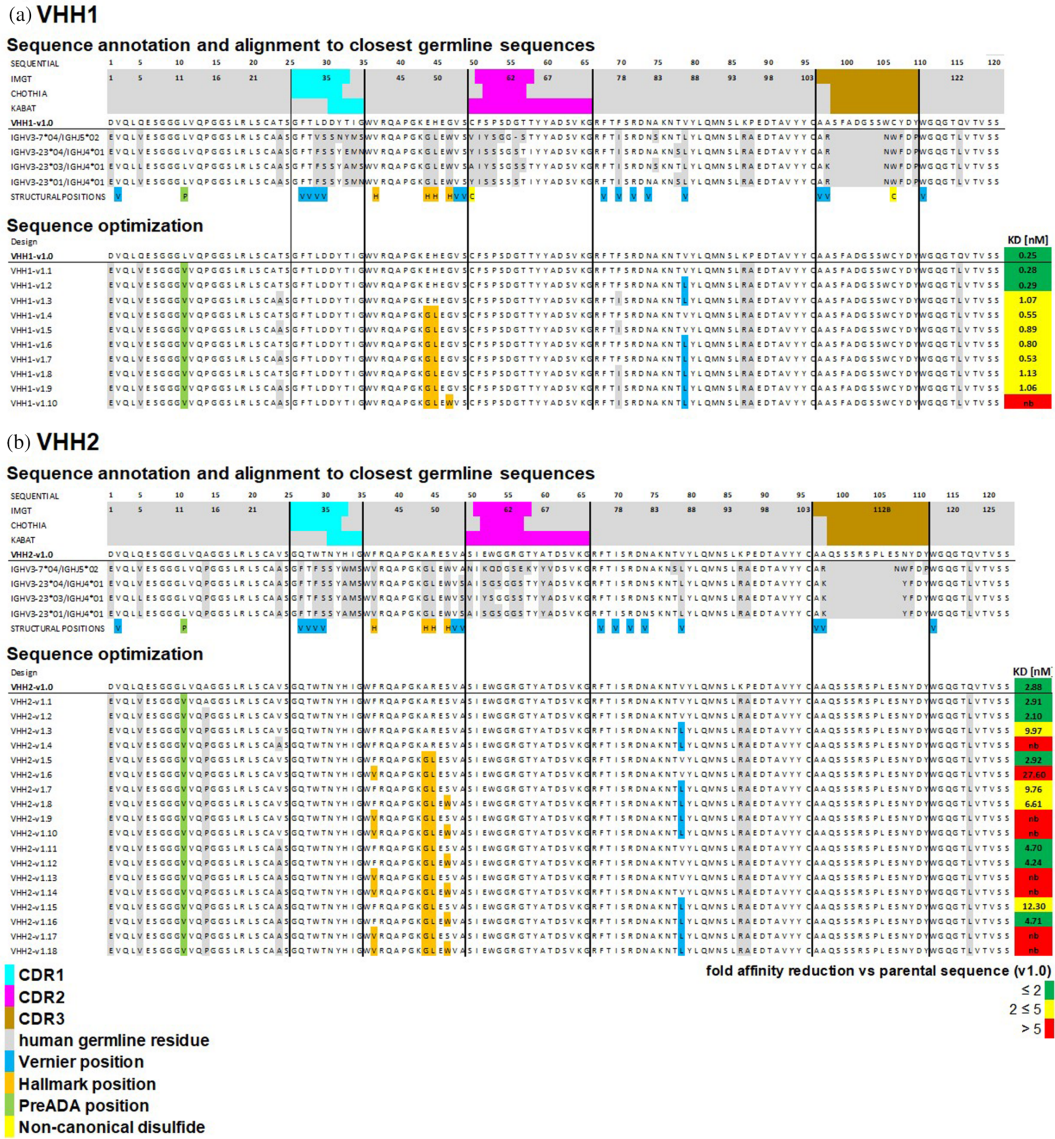
(a) VHH1 and (b) VHH2 sequence annotations and alignment to the most similar germline sequences. The first three rows indicate CDR1‐3 (cyan, magenta, brown) residues according to IMGT, Chothia and Kabat nomenclature in green. The fourth row shows the sequence of parental VHHs and the following four rows the alignment to the most similar germline sequences. Amino acid differences to the most similar germline sequence are indicated in gray. The last row shows residues in different key structural positions: Vernier positions (indicated in blue), Hallmark positions (orange), one position known to be essential for binding to preADAs (green) and non‐canonical cysteines (yellow). Sequence optimization: Designed variants towards increased human‐likeness in the framework regions. Mutations that have been introduced in key structural positions are colored accordingly. For straightforward sequence activity relationship (SAR) analysis, the KD values (Table 1) are added at the end of each designed sequence and are complemented with a green to red coloring based on the affinity reduction compared to the parental sequence.

The discovery of VHH1 and VHH2 was previously detailed (Jumper et al., 2021). In brief, it involved llama immunization followed by yeast surface display‐based antibody selection and in‐depth characterization of candidates in terms of NK cell redirection efficiencies. We utilized our internal pipeline, termed *Sequence Assessment Using Multiple Optimization Parameters* (SUMO) (Evers et al., 2023), to assess and annotate the sequences (Figures 1 and S1 and Section 5 for details).

In a process akin to the humanization workflow described by Sulea (Sulea, 2022), we engrafted the CDR1‐3 regions of the parental camelid sequences (VHH1‐v1.0 and VHH2‐v1.0) onto modified versions of their closest human germline sequences. In the first variant, residues in Vernier or Hallmark positions remained unchanged, while Leu12 (according to IMGT numbering) was replaced by valine to mitigate reactivity to pre‐existing anti‐drug antibodies (preADAs) (Johansson et al., 2023; Lin et al., 2021). From this sequence (v1.1 in Figure 1), positions assumed to interact with CDRs, including Vernier and Hallmark residues, were incrementally humanized to increase similarity to the closest human germline sequence. These modifications were introduced step‐by‐step or in combinations (VHH1‐v1.2–VHH1‐v1.10 in Figure 1a and VHH2‐v1.2–VHH2‐v1.18 in Figure 1b), to optimize the balance between human‐likeness and antigen binding after a single Design, Make, Test, Analyze (DMTA) cycle. Subsequently, all designed variants underwent in silico sequence assessment using SUMO (see results in Figure S1).

### 2.2 Sequence production, experimental profiling, and sequence activity relationship (SAR) analysis

Since VHHs are commonly used as paratope‐building blocks of more complex antibody structures, we produced generated sequences as bispecific antibody derivatives, referred to as VHH SEEDbodies (Davis et al., 2010) (see Section 5 for details). Expression yields after protein A purification were in the double to triple digit milligram per liter range, generally indicating adequate profiles for transient production (Table 1). For all molecules within the VHH2 series, purities as determined by analytical size exclusion chromatography (SEC) post single step purification were above 85% target peak (Table 1). Interestingly, while for VHH2 there was no correlation between the degree of humanization and purities, for VHH1 we observed a trend towards lower purities for all molecules beyond VHH1‐v1.3.

**TABLE 1:** Binding affinity and analytical data for the parental and humanized sequence variants of VHH1 and VHH2.

	KD (nM)	*K*_on_ (1/Ms)	*K*_off_ (1/s)	*E*max_100nM_ (nm)	*R* ^2^	Yield (mg/L)	SEC purity (%)
VHH1‐v1.0	0.2	1.1E+06	2.7E‐04	0.13	0.99	66.5	87.2
VHH1‐v1.1	0.3	1.0E+06	2.9E‐04	0.13	0.99	58.8	89.5
VHH1‐v1.2	0.3	1.1E+06	3.2E‐04	0.12	1.00	41.9	85.3
VHH1‐v1.3	1.1	8.8E+05	9.4E‐04	0.12	0.99	38.3	85.4
VHH1‐v1.4	0.5	1.1E+06	6.2E‐04	0.12	0.99	86.3	76.8
VHH1‐v1.5	0.9	1.1E+06	9.7E‐04	0.10	0.98	87.3	73.0
VHH1‐v1.6	0.8	1.1E+06	8.7E‐04	0.14	0.99	74.1	63.8
VHH1‐v1.7	0.5	1.0E+06	5.5E‐04	0.09	0.99	80.7	65.0
VHH1‐v1.8	1.1	1.1E+06	1.3E‐03	0.10	0.99	70.0	73.0
VHH1‐v1.9	1.1	8.7E+05	9.3E‐04	0.10	0.98	68.4	69.8
VHH1‐v1.10	nb	nb	nb	nb	nb	85.2	64.4
VHH2‐v1.0	2.9	2.3E+05	6.6E‐04	0.17	1.00	98.2	89.1
VHH2‐v1.1	2.9	2.7E+05	7.9E‐04	0.18	1.00	104.7	89.5
VHH2‐v1.2	2.1	3.5E+05	7.4E‐04	0.15	1.00	120.2	89.5
VHH2‐v1.3	10.0	9.3E+04	9.3E‐04	0.13	1.00	43.1	97.3
VHH2‐v1.4	nb	nb	nb	nb	nb	26.1	100.0
VHH2‐v1.5	2.9	3.8E+05	1.1E‐03	0.11	0.99	86.6	95.7
VHH2‐v1.6	27.6	4.6E+04	1.3E‐03	0.07	0.98	77.6	94.0
VHH2‐v1.7	9.8	1.1E+05	1.1E‐03	0.10	0.99	63.1	96.9
VHH2‐v1.8	6.6	1.3E+05	8.4E‐04	0.10	0.99	109.9	94.4
VHH2‐v1.9	nb	nb	nb	nb	nb	37.5	98.1
VHH2‐v1.10	nb	nb	nb	nb	nb	42.3	97.9
VHH2‐v1.11	4.7	1.6E+05	7.4E‐04	0.16	0.99	100.1	93.5
VHH2‐v1.12	4.2	1.3E+05	5.5E‐04	0.14	0.99	118.6	93.3
VHH2‐v1.13	nb	nb	nb	nb	nb	98.6	95.8
VHH2‐v1.14	nb	nb	nb	nb	nb	82.1	95.9
VHH2‐v1.15	12.3	5.8E+04	7.2E‐04	0.07	0.99	21.5	99.1
VHH2‐v1.16	4.7	8.7E+04	4.1E‐04	0.11	0.99	86.1	97.8
VHH2‐v1.17	nb	nb	nb	nb	nb	18.0	100.0
VHH2‐v1.18	nb	nb	nb	nb	nb	91.9	97.1

*Note*: *K*_on_ is the rate constant of association, while *K*_off_ is the rate constant of dissociation. Emax is the maximum interference pattern shift that was observed for a sample in the experiment, reflecting the maximum binding capacity. *R* ^2^: fit quality to the experimental BLI data.

To facilitate SAR analysis, Figure 1 displays the experimental KD values alongside the designed sequences using a color scale (green to yellow to red) that represents the affinity loss compared to the parental sequence. Due to the experimental noise of the BLI technique and typical batch‐to‐batch variabilities, here we consider binding affinity reduction above a factor of 2 as relevant.

All VHH1 variants, except v1.10, exhibited strong binding affinity, with a maximal reduction by 4.6‐fold. Humanization outside CDRs, Vernier and Hallmark positions (VHH1‐v1.1, see Figure 1a and Table 1) resulted in minor reduction of binding affinity. SAR analysis revealed VHH1's tolerance to humanization of Vernier zone residue V87L (IMGT nomenclature). However, humanization of Vernier zone residue F78I led to a slight reduction of binding affinity (~2‐fold). VHH1's Hallmark signature VEHG allowed for humanization of positions 49 and 50 (E49G, H50L) without major affinity reduction, but mutation G52W was not tolerated. In summary, VHH1 could be sequence‐optimized towards a considerably high degree of human‐likeness within the framework region without significant reduction of binding affinity. Notably, VHH1 carries two non‐canonical cysteines in position 55 and 114 (CDR3) that form a disulfide bond.

Also, for VHH2, SAR analysis revealed that humanization of non‐CDRs, non‐Vernier and non‐Hallmark zone residues did not affect binding affinity. In contrast to VHH1, humanization of Vernier zone residue V87L reduced binding affinity by a factor of ≤5 when comparing matched molecular pairs (MMPs). VHH2 carries the Hallmark signature *FARS* (positions 42, 49, 50, 52 according to IMGT nomenclature). SAR and MMP analysis unveiled that Hallmark residues Ala49, Arg50 and Ser52 (the latter in contrast to VHH1) can be mutated into the humanized residues without impact on binding affinity (Figure 1b and Table 1). Peculiarly, mutation S52W even showed a slight positive impact on binding affinity (VHH2‐v1.7 vs. VHH2‐v1.8, VHH2‐v1.11 vs. VHH2‐v1.12 and VHH2‐v1.15 vs. VHH2‐v1.16). However, the (additional) mutation of Hallmark residue F42V consistently resulted in a considerable reduction of binding affinity (VHH2‐v1.5 vs. VHH2‐v1.6, VHH2‐v1.7 vs. VHH2‐v1.9, VHH2‐v1.8 vs. VHH2‐v1.10, VHH2‐v1.11 vs. VHH2‐v1.13, VHH2‐v1.12 vs. VHH2‐v1.14, VHH2‐v1.15 vs. VHH2‐v1.17 and VHH2‐v1.16 vs. VHH2‐v1.18). Overall, variant VHH2‐v1.16 struck the best compromise between binding affinity (KD = 4.7 nM compared to KD = 2.9 nM for the parental version) and degree of humanization within the framework region, even after simultaneous humanization of 5 Vernier zone or Hallmark residues.

### 2.3 Crystal structure and binding hypothesis generation using AlphaFold2

**FIGURE 2:**
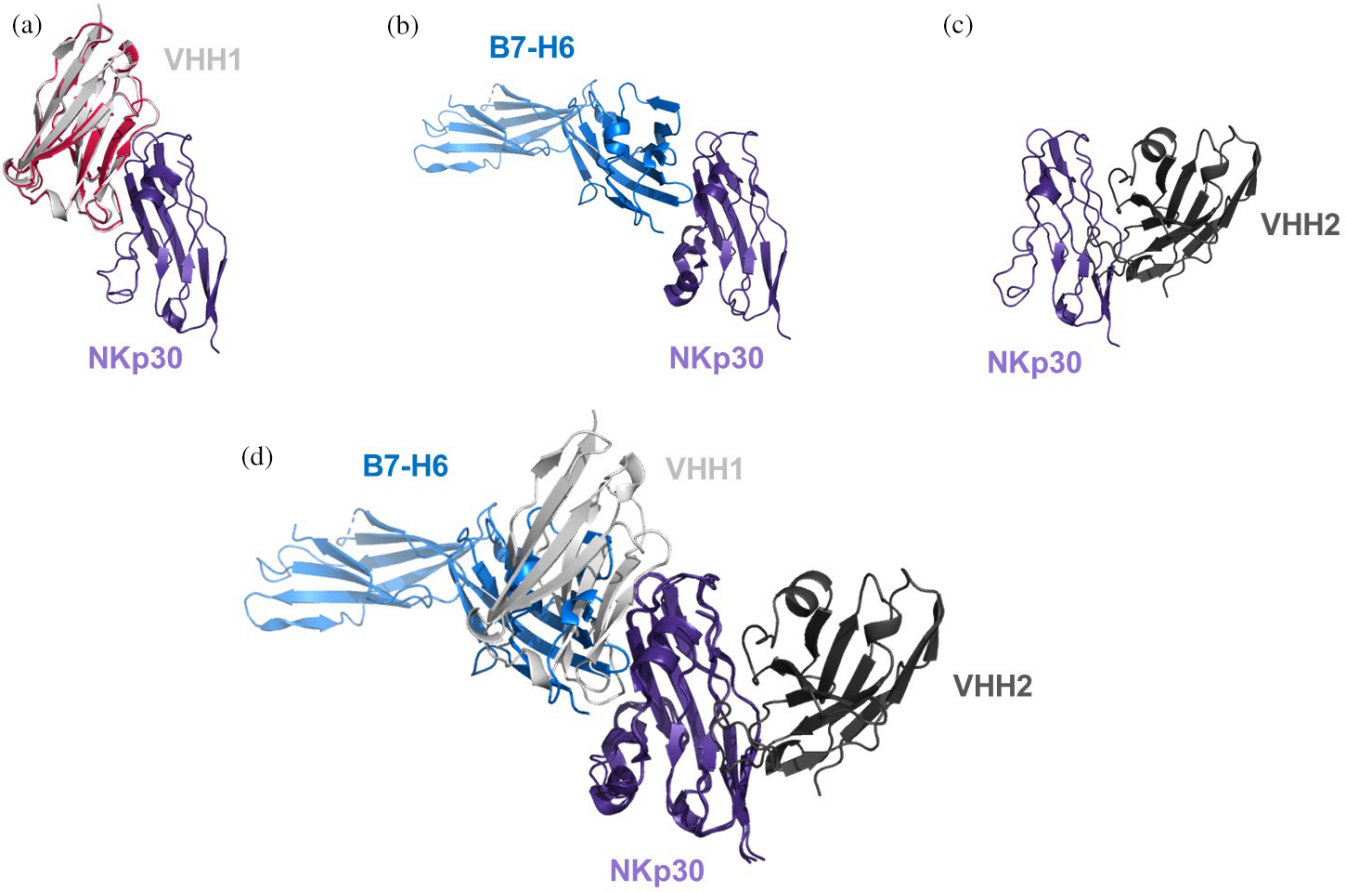
Crystal structures and AlphaFold2 generated binding models of NKp30 with different ligands. (a) X‐ray structure of apo‐VHH1 (shown in red, PDB code 9FXF), superimposed in the AlphaFold2 generated VHH1‐NKp30 complex, reveals that the VHH1 apo structure matches the VHH1 conformation of the AlpaFold2 generated VHH1‐NKp30 complex. (b) Binding mode of the B7‐H6–NKp30 complex (PDB code 3PV6). (c) X‐ray structure of the VHH2‐NKp30 complex (PDB code 9FWW). (d) Superimposition of VHH1‐, VHH2‐, and B7‐H6‐binding modes against NKp30 reveals that the epitopes of B7‐H6 and VHH1 are overlapping, while VHH2 is binding to a distinct epitope.

During the process of sequence production and testing, attempts were made to crystallize both parental VHH domains in complex with the extracellular domain of human NKp30. A structure for the NKp30‐VHH2 complex was successfully obtained (PDB code 9FWW, resolution: 1.84 Å, Figure 2c). Despite efforts, a high‐resolution complex for NKp30‐VHH1 could not be attained. However, a high‐resolution structure for uncomplexed VHH1 was obtained (PDB code 9FXF, colored red in Figure 2a). Using Colabfold implementation of Alphafold2 multimer (Jumper et al., 2021; Mirdita et al., 2022), a binding hypothesis for the NKp30‐VHH1 complex was generated (Figure 2a). Remarkably, the predicted binding conformation of VHH1 closely resembles the experimentally observed x‐ray conformation of uncomplexed VHH1 (Cα rmsd: 0.66 Å, see alignment in Figure 2a). Additionally, the crystal structure of NKp30 bound to its ligand B7‐H6 (pdb 3 pv6) (Li et al., 2011) is depicted in Figure 2b. Consistent with epitope binning experiments from our previous study (Klausz et al., 2022), according to AlphaFold2 prediction, VHH1 binds to an overlapping epitope with B7H6, while VHH2 targets a distinct epitope.

### 2.4 Structure–activity relationship analysis and MD simulations

**FIGURE 3:**
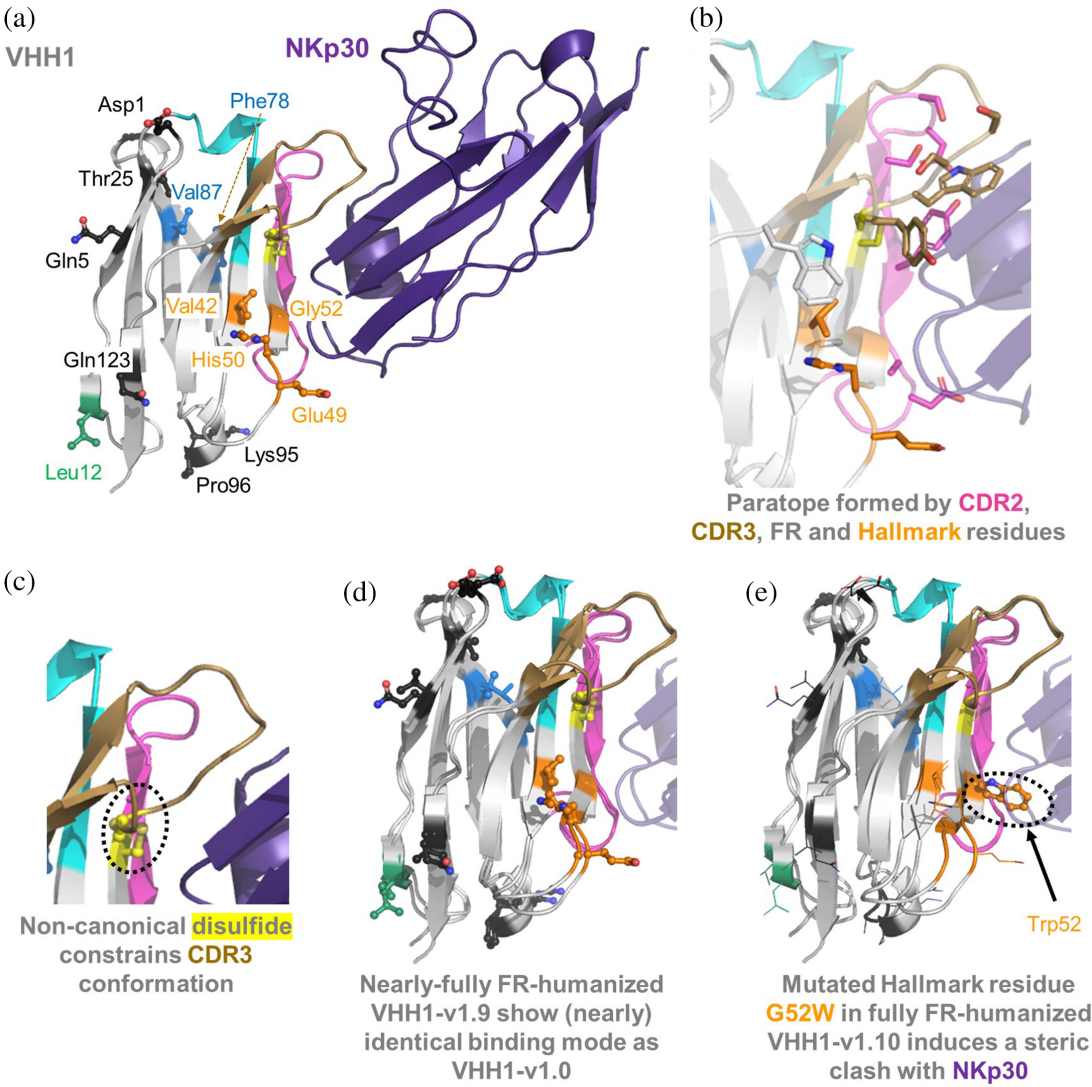
(a) AlphaFold2 predicted VHH1‐NKp30 complex. (b) Visualization of VHH1 paratope residues (within 4.5 Å of NKp30). (c) Depiction of the non‐canonical disulfide bond that covalently links CDR3 to Cys55. (d) 3D‐alignment of AlphaFold2 predicted binding modes of parental VHH1‐v1.0 and humanized VHH1‐v1.9. (e) 3D‐alignment of parental VHH1‐v1.0 and fully framework‐humanized VHH1‐v1.10 indicates a possible steric clash of humanized Hallmark residue Trp52 with NKp30. Key residues that were subjected to humanization are indicated in the same color coding as in Figure 1.

*VHH1*: The AlphaFold2 predicted paratope of VHH1 comprises residues from CDR2, CDR3, the framework region (Trp118), and all four residues of the Hallmark signature (Figure 3a,b). According to the predicted complex, there are no direct intramolecular interactions between any Hallmark and a CDR3 residue. As described above, humanization of Hallmark residues 49 and 50 did not significantly affect the binding affinity of VHH1. Structural analysis of the predicted complex suggests no specific charge‐assisted interactions of Hallmark residues with NKp30, rationalizing the tolerance of the simultaneous E49G & H50L Hallmark residue mutations. The AlphaFold2 predicted complex of VHH1‐v1.9, with nearly complete humanization of the framework region, including Hallmark residues 49 and 50, exhibits an almost identical structural fold and binding mode compared to parental VHH1‐v1.0 (Figure 3d). However, full humanization of the VHH1 framework region (VHH1‐v1.10) results in complete loss of antigen binding. A 3D alignment of the predicted binding modes of VHH1‐v1.0 and VHH1‐v1.10 (Figure 3e) suggests that this loss is due to the G52W Hallmark residue mutation, which induces unfavorable steric interactions with NKp30. Notably, VHH1 possesses Cys107 in CDR3, forming a non‐canonical disulfide bond with Cys55. Further analysis (below) reveals that non‐canonical disulfides, such as this, are commonly observed in camelid derived VHHs and serve as general mechanism to constrain CDR3 in the bioactive conformation.

**FIGURE 4:**
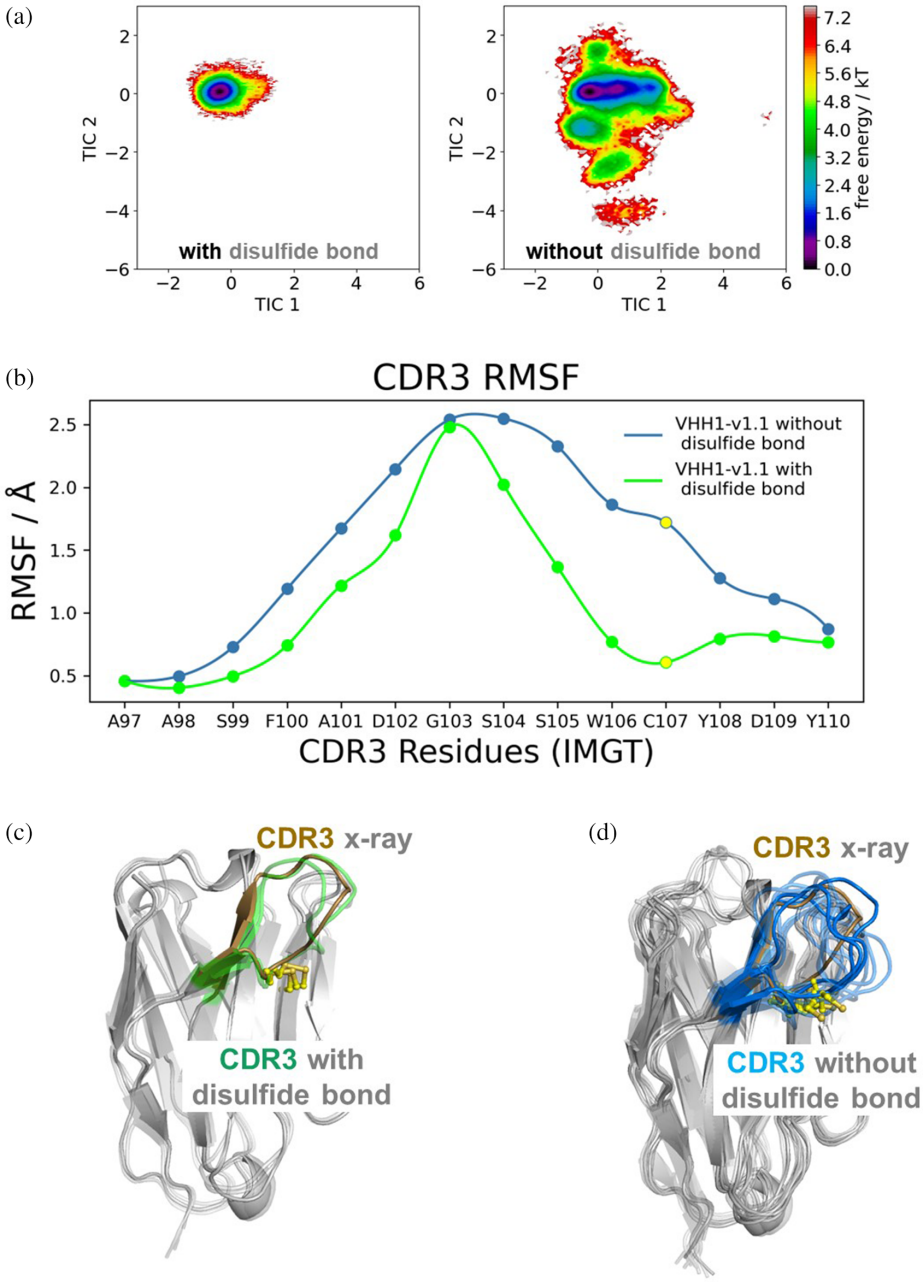
Non‐canonical disulfide bridge substantially stabilizes the CDR3 loop in the binding competent conformation. (a) Free energy landscape of the CDR3 loop for VHH1‐v1.0 with and without disulfide bridge shows a substantial increase in conformational diversity. (b) Root mean square fluctuation (RMSF) for CDR3 residues for the MD simulations of VHH1‐v1.1 with and without the disulfide bridge between residues Cys55 and Cys107 (highlighted in yellow). Structural visualization of the conformational ensembles obtained from the clustering analysis using the same RMSD distance cut‐off criterion (2.5 Å) for VHH1‐v1.1 (c) with and (d) without disulfide bond between non‐canonical cysteines Cys55 and Cys107, aligned to the x‐ray structure of VHH1‐v1.0 (PDB code 9FXF), illustrating that the formation of the cysteine bridge stabilizes the binding competent CDR3 conformation.

To further elucidate on the role of Hallmark residues and the non‐canonical disulfide on conformational stability of VHH1, we performed MD simulations on VHH1‐v1.1 with and without the disulfide bridge. Figure 4 shows the free energy landscape and root mean square fluctuation (RMSF) analysis for the CDR3 loop of VHH1‐v1.1 for both variants, demonstrating the substantial increase in CDR3 loop flexibility and conformational diversity in the absence of the disulfide bridge. This increase in variability of CDR3 further emphasizes the important role of the disulfide bridge in stabilizing the CDR3 loop and consequently the paratope.

**FIGURE 5:**
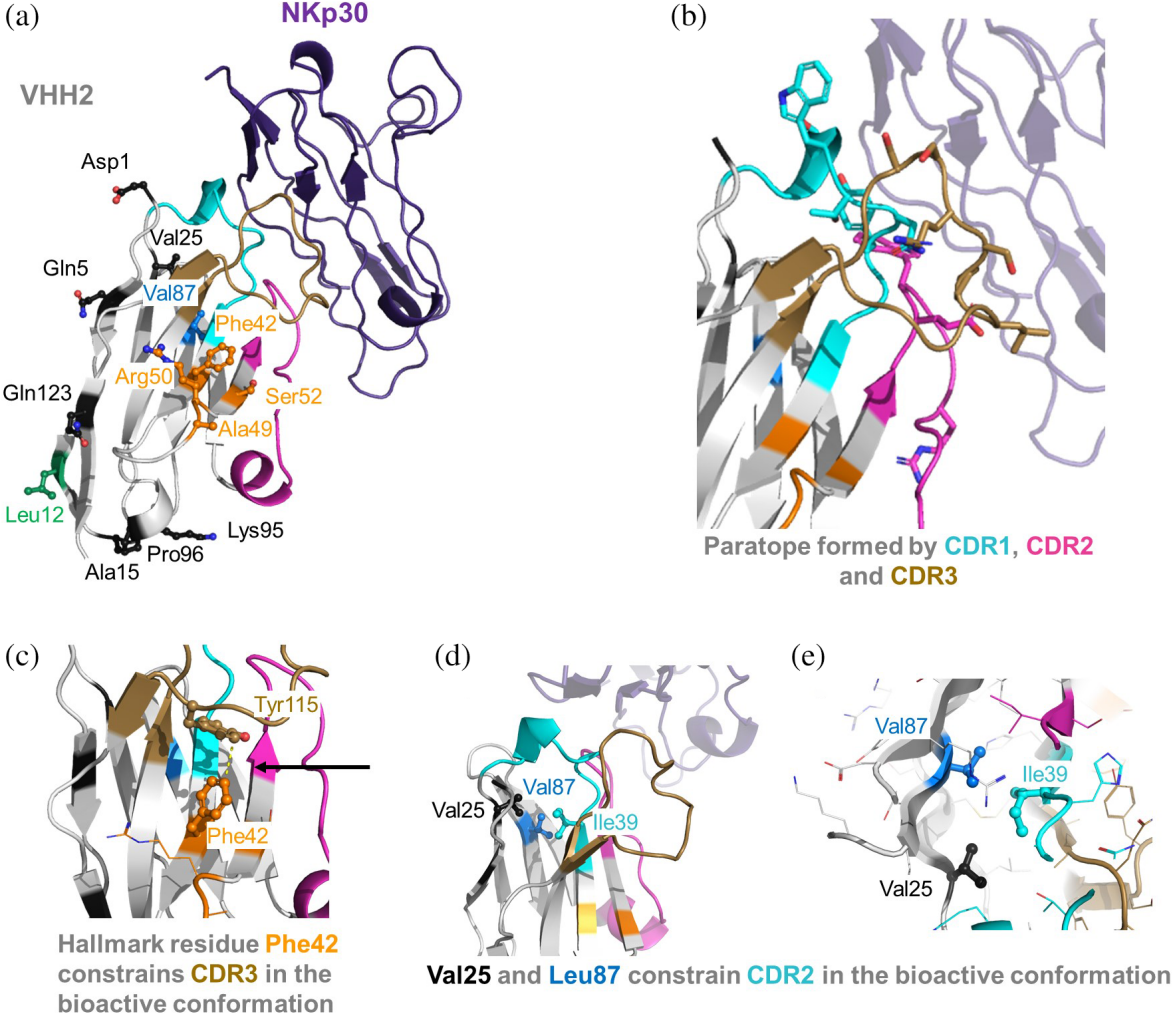
(a) X‐ray structure of the VHH2‐NKp30 complex (PDB code 9FWW). (b) Visualization of VHH2 paratope residues (within 4.5 Å of NKp30). (c) Depiction of an intramolecular interaction between Hallmark residue Phe42 and CDR3 residue Tyr115 that seems essential for positioning CDR3 in its bioactive conformation. (d, e) visualize a network of hydrophobic residues between Val25, Ile39 and Val87 that seem to be essential to position CDR2 in the bioactive conformation. Key residues that were subjected to humanization are indicated in the same color coding as in Figure 1.

**FIGURE 6:**
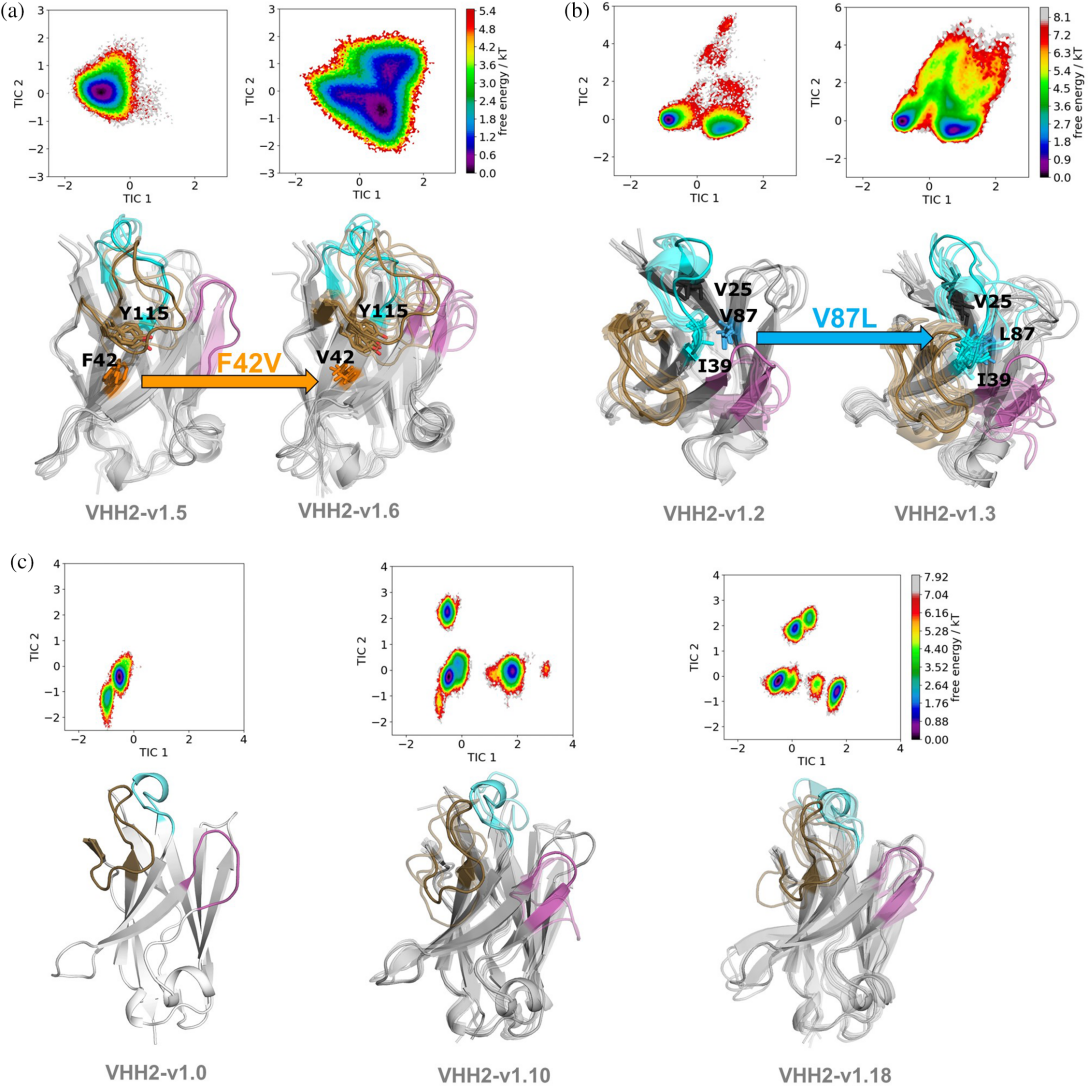
Structural and dynamic characterization of Hallmark mutations suggesting conformational entropy as critical determinant for antigen‐recognition. (a) Mutation F42V destabilizes the CDR3 loop, resulting in a bigger conformational space and a higher number of low populated states. The higher conformational diversity of the CDR3 loop is also reflected in the broader conformational ensemble. (b) In addition to directly stabilizing the CDR loops, the Hallmark residue mutation V87L reveals a conformational rearrangement of the CDR2 and CDR1 loops to accommodate the bulkier leucine sidechain. (c, d) Free energy surface of the paratope for VHH2‐v1.0 compared with VHH2‐v1.10 and VHH2‐v1.18. Both VHH2‐v1.10 and VHH2‐v1.18 are non‐binders and reveal a substantially increased conformational diversity reflected in a broader conformational ensemble, accompanied by a substantial population shift towards three equally lower populated states in contrast to one dominant state corresponding to the binding competent state for VHH2‐v1.0.

*VHH2*: visual analysis of the VHH2‐NKp30 complex (PDB code 9FWW) reveals that VHH2, in contrast to VHH1, interacts with NKp30 by engaging residues from CDR1‐3 (Figure 5a,b). While the Hallmark residues do not directly interact with the antigen, the humanization of F42V consistently resulted in considerable reduction of binding affinity. Figure 5c illustrates that Phe42 forms a T‐shaped aromatic pi interaction with Tyr115 (CDR3), obviously essential for positioning and stabilizing CDR3 in its bioactive conformation. The stabilizing role of Phe42 is further emphasized by the MD simulations when comparing the conformational diversity of VHH2‐v1.5 (KD = 2.9 nM) with its F42V variant VHH2‐v1.6 (KD = 27.6 nM). We find a substantial increase in variability for the CDR3 loop resulting from the destabilization of the “T‐like” stacking and hydrophobic interactions between Phe42 and Tyr115, which is reflected also in a broader conformational space and in a higher number of structural clusters as measure of conformational diversity (Figure 6a).

In line with previous literature suggestions (Vincke et al., 2009) and structural observations indicating minimal intermolecular or intramolecular interactions in VHH2, humanization of Hallmark residues 49 and 50 (A49G & R50L) does not impact binding affinity. Unlike VHH1, VHH2 tolerates the humanization of Hallmark residue 52 (S52W), consistent with the structural observation that Ser52 is distant from the NKp30 epitope. Humanization of Vernier zone residue V87L results in a 4.8‐fold affinity reduction in VHH2‐v1.3 compared to VHH2‐v1.2. Comparative analysis of MD simulations for both variants suggests that the V87L mutation induces a significant overall VHH conformational change, involving the CDR1‐3 paratope region, but especially affecting CDR2 and CDR1 loops (Figure 6b), to accommodate the Leu mutation. Interestingly, the affinity reduction is less pronounced in the V87L matched molecular pairs VHH2‐v1.15 vs. VHH2‐v1.11 (factor 2.6) or VHH2‐v1.16 vs. VHH2‐v1.12 (factor 1.1). Figure 5d shows that Val87 participates in a network of hydrophobic interactions involving Val25 (FR1) and Ile39 (CDR1). Notably, simultaneous mutations of V87L and V25A (as in VHH2‐v1.11and VHH2‐v1.12) results in similar hydrophobic packing, probably maintaining CDR1 in its bioactive conformation. In summary, VHH2's framework region can be humanized to a high degree. Compared to the parental sequence VHH2‐v1.0, VHH2‐v1.16 exhibits almost complete humanization of the FR (except for Hallmark residue Phe42 and the intentionally introduced anti‐preADA mutation L12V), with only a 1.6‐fold affinity reduction.

To unravel the mechanistic effects of multiple mutations on the conformational diversity of the CDRs, we performed MD simulations of binders and non‐binders. Comparing VHH2‐v1.0 with VHH2‐v1.10 and VHH2‐v1.18 (Figure 6c) suggests that conformational entropy is a critical determinant for antigen binding as it strongly influences the shape of the paratope. While VHH2‐v1.0 reveals one dominant state in solution, VHH2‐v1.10 and VHH2‐v1.18 show multiple relatively highly populated paratope states. A structural overlay of these states highlights that not only the CDR3 loop but also CDR1 and CDR2 show distinct conformational states, compared to the bioactive conformational state observed for VHH2‐v1.0.

In summary, both VHHs can be optimized to achieve a significant degree of human‐likeness. Notably, the roles of Vernier zone and Hallmark regions differ considerably between VHH1 and VHH2. While the humanization of Vernier zone residue V87L was tolerated in VHH1, a slight affinity reduction was observed in VHH2. In VHH2, the Hallmark region does not contribute to the paratope but appears crucial for constraining CDR3 into its bioactive conformation. Conversely, in VHH1, the AlphaFold2‐predicted complex suggests that the Hallmark residues are integral to the NKp30 paratope rather than directly interacting with or stabilizing CDR3. Interestingly, conformational stabilization of CDR3 is facilitated by the presence of the non‐canonical disulfide in VHH1. For both VHHs, the Hallmark region could be partially, but not fully humanized. In VHH1, the humanization of G52W resulted in loss of binding affinity due to predicted steric clashes with NKp30, while in VHH2 the mutation F42V impacts binding affinity, presumably due to de‐stabilization of the CDR3 bioactive conformation.

### 2.5 Unraveling the significance of Hallmark residues and non‐canonical disulfides in VHHs

In this section, we undertake a systematic investigation into the role of Hallmark residues and non‐canonical disulfide bonds in huge VHH datasets. As previously discussed, and verified in this study, VHHs employ variations in Hallmark positions and non‐canonical cysteine bridges as additional mechanisms to compensate for the absence of a light chain. These adaptations serve to increase paratope size and diversity, as well as to stabilize the bioactive conformation. We investigate camelid germline sequences, analyze camelid VHH NGS repertoires, and scrutinize PDB structures to unravel the functional significance of these structural VHH features.

#### 2.5.1 Llama and alpaca V‐genes exhibit variability in Hallmark motifs and contain unpaired cysteines

**FIGURE 7:**
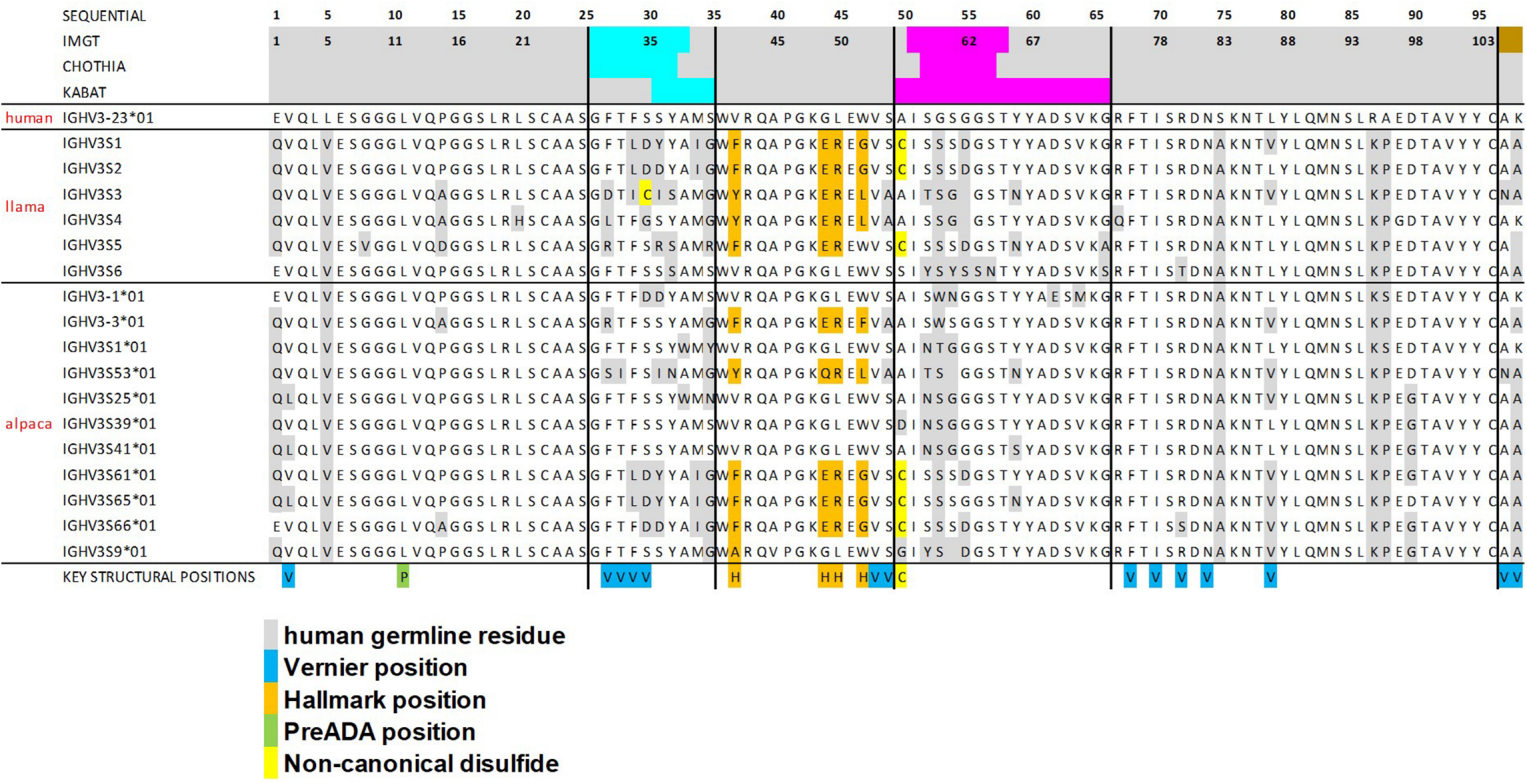
Sequence alignment of most prevalent V‐genes from llamas or alpacas. For comparison, the sequences of human IGHV3‐23*01 as generally recommended template for most VHH humanization campaigns (Sulea, 2022), is provided. Key residues are indicated in the same color coding as in Figure 1.

Comparison of all llama germline V‐gene sequences from IMGT (https://www.imgt.org) and the most prevalent alpaca germline V‐genes (Tu et al., 2020) highlights the diversity of Hallmark motifs encoded in these sequences (Figure 7). Moreover, the presence of unpaired non‐canonical cysteines in four out of six llama and three out of 11 alpaca V‐gene sequences suggests the inherent capacity for forming non‐canonical disulfide bonds with somatically introduced cysteines in CDR3, contributing to enhanced conformational stability (Conrath et al., 2003).

#### 2.5.2 The exploration of camelid VHH NGS repertoires and PDB structures uncovers significant diversity in Hallmark motifs and a prevalent utilization of non‐canonical disulfide bonds

**FIGURE 8:**
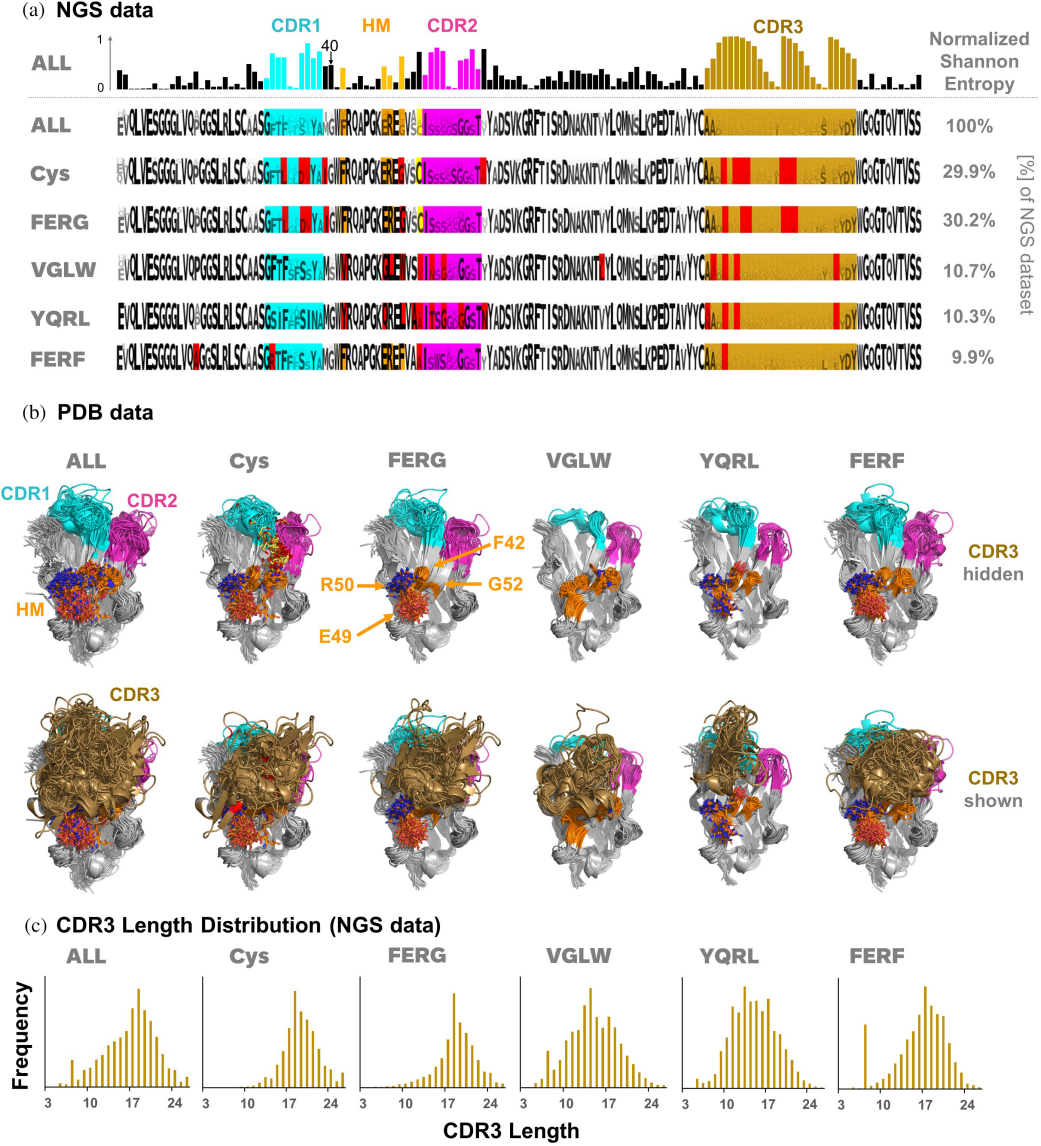
Visualization of (a) sequence and (b) structure diversity of VHHs as observed in NGS and PDB data. (a) Normalized Shannon entropy of residues along the VHH sequence (top row) and sequence logos of the entire VHH NGS data set (ALL) and for specific subsets, together with their percentages observed in the dataset. Positions colored in red indicate that the most prevalent residue in this position is different from the most prevalent residue in the entire dataset (ALL). (b) 3D‐alignment of PDB structures, based on the same subset definitions as for the NGS subsets. For clarity, CDR3 is hidden in the upper row and shown in the lower row. (c) CDR3 length distribution over the entire NGS data set and for the specific subsets. Key residues are indicated in the same color coding as in Figure 1. CDR and FR definitions are based on the IMGT nomenclature.

To delve deeper into the functional significance of Hallmark motifs and non‐canonical cysteine bridges in VHHs, we conducted a follow‐up investigation, building upon previous studies (Bahrami Dizicheh et al., 2023; Deszyński et al., 2022; Kuroda & Tsumoto, 2023; Murakami et al., 2022). Our examination involved the analysis of Hallmark motifs and non‐canonical cysteine bridges across existing VHH NGS datasets and 642 PDB structures. Deszynski et al (Deszyński et al., 2022) reported that FERF, FERG, VGLW, and YQRL are the most prevalent Hallmark motifs across seven different NGS studies, collectively accounting for 61.1% of the dataset sequences. These motifs were also most prominent in the investigated PDB dataset (FERF: 157, VGLW: 121, FERG: 103, YQRL: 52). For a systematic sequence analysis of camelid VHH repertoires, we retrieved sequences from the NGS data provided by Deszynski et al (2022), processed them as described in Section 5, and generated logo plots for the entire data set (ALL) and specific subsets representing sequences with additional non‐canonical disulfide bridges (Cys) or the most prevalent Hallmark motifs (FERG, VGLW, YQRL, FERF, see Figure 8a).

Key observations from our analysis of camelid VHH NGS data include: First, a substantial presence of non‐canonical disulfide bonds with CDR3 residues was found in 29.9% of all sequences, with cysteine at position 55 being the most frequent partner for disulfide formation with CDR3. This cysteine position is particularly favored in combination with the FER*G* Hallmark motif, a pattern consistent with its prevalence in camelid germline sequences (Figure 7). Next, the normalized Shannon entropy analysis across the entire dataset (ALL, Figure 8a, upper row) showed the highest variability of amino acids within and around the CDRs and within the Hallmark motifs, with residue 52 exhibiting the highest variability among Hallmark positions. Preferred combinations of sequence motifs observed in the NGS data are already encoded in camelid V‐genes (Figure 7), indicating evolutionary conservation and potential functional and structural significance.

Figure 8b presents the 3D alignment of 642 VHH structures extracted from the PDB (see Section 5 for details), covering all structures (ALL) and corresponding subsets (Cys, FERG, VGLW, YQRL, and FERF) analyzed in tandem with the NGS data. Our structural analysis yielded several insights. There is a pronounced structural conservation of VHH backbones, particularly in the FR. Hallmark residues, by spatial arrangement, predominantly interact with CDR3 rather than CDR1 or CDR2. In many cases, CDR3 conformation is effectively sandwiched by interactions with CDR1 and CDR2 on one side and Hallmark residues on the other. The sidechains of Hallmark residues exhibit different surface geometries and polarities (Figure 8b, upper row), fostering differential interactions with varied CDR3 geometries (Figure 8b, lower row). Consistent with the NGS data, non‐canonical cysteine residues within the framework regions are predominantly situated at position 55 (N‐terminally to CDR2 according to IMGT nomenclature), serving as a covalent anchor to accommodate and constrain diverse CDR3 conformations (Figure 8a,b). Both germline sequences and NGS data show a preferential occurrence of a non‐canonical disulfide bond involving Cys55 in combination with the FERG Hallmark motif (Figures 7 and 8a). Hallmark residue 52, identified as the most diversified Hallmark residue according to Shannon entropy analysis, assumes a bulky form in VGL*W*, YQR*L*, and FER*F*, whereas it is small in FER*G*, where the proximal non‐canonical Cys residue acts as an alternative anchor and conformational stabilizer of CDR3. Additionally, CDR3 conformations in sequences with the YQRL Hallmark signature exhibit less structural diversity compared to other motifs. Recent studies link the occurrence of Tyr42 in *Y*QRL with an extended CDR3 conformation in VHHs, contrasting with the more kinked CDR3 conformation induced by other Hallmark motifs (Bahrami Dizicheh et al., 2023; Kuroda & Tsumoto, 2023), resulting in different interactions with antigens. Visual analysis of the aligned PDB structures (Figure 8b, lower row) highlights that Hallmark residues 49 and 50 are frequently solvent‐exposed. If these residues are polar (as in F*ER*F, F*ER*G, and Y*QR*L), they might enhance VHH domain solubility and/or serve as additional specific interaction points with antigens.

Finally, Figure 8c depicts the observed CDR3 length distribution in the NGS dataset for all sequences (ALL) and the different subsets (Cys, FERG, VGLW, YQRL and FERF). The average CDR3 lengths over the entire NGS data set (ALL) is 16.6 residues. Notably, VHHs carrying the FERG Hallmark motif (average length: 18.4) and/or an additional non‐canonical disulfide bond (19.0) tend to exhibit longer CDR3 sequences compared to the other Hallmark‐based subsets (VGLW: 14.4, YQRL: 14.6, FERF: 16.3). Such longer CDR3 sequences facilitate the incorporation of additional secondary structures, enabling the VHH to target a high diversity of epitope geometries, as discussed in previous studies (Kuroda & Tsumoto, 2023), again confirming that these germline‐encoded combinations of Hallmark motifs and non‐canonical disulfides contribute to the expanded antigen‐binding repertoire of VHHs.

#### 2.5.3 Analysis of pairwise correlations and normalized mutual information (NMI) on camelid VHH NGS repertoires and an extended Hallmark definition

**FIGURE 9:**
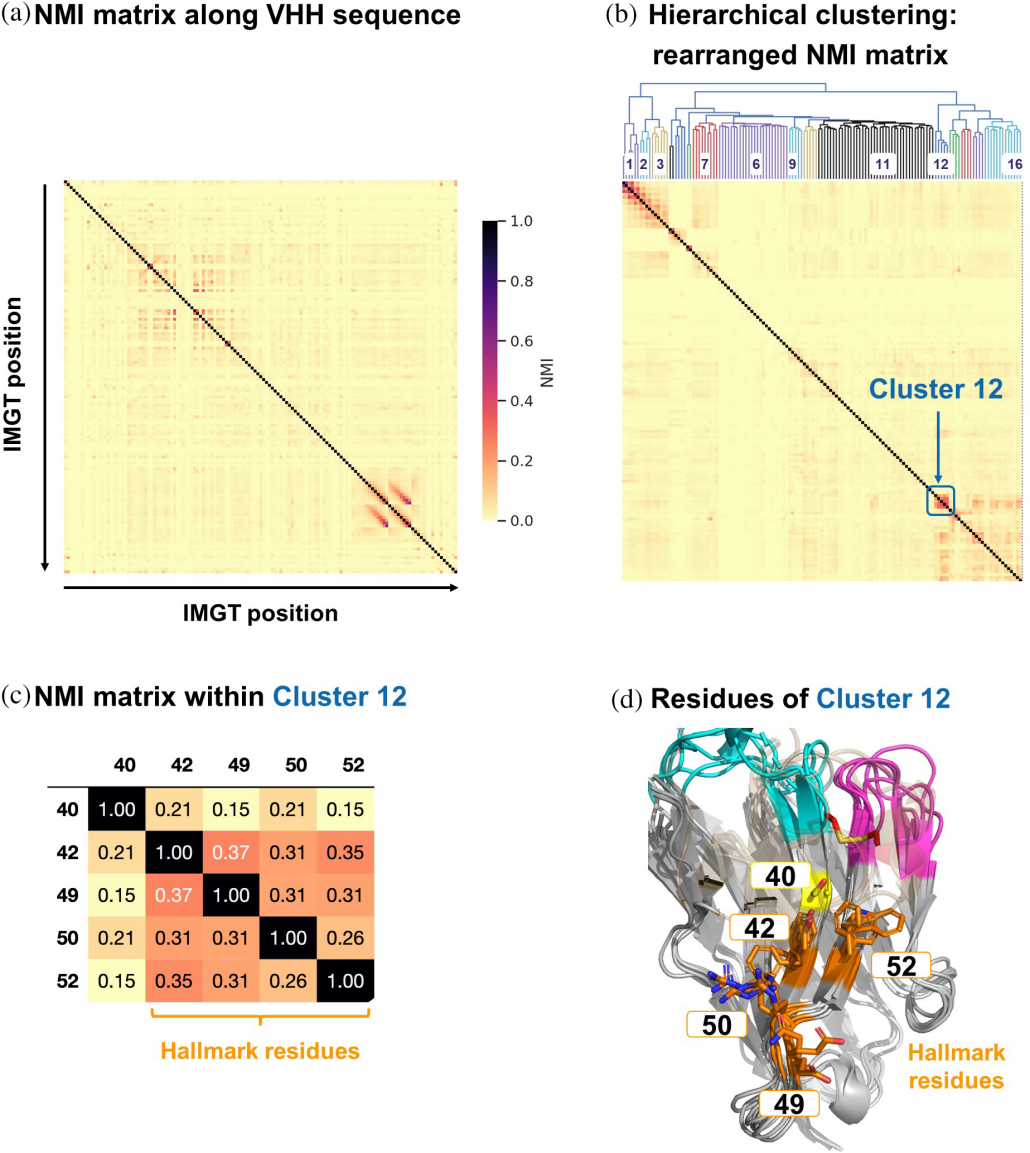
(a) NMI matrix based on normalized Shannon entropies along the VHH sequence (in IMGT numbering). (b) Rearranged NMI matrix, including the dendrogram associated with the hierarchical clustering and the assigned cluster IDs. Cluster 12 is indicated in the matrix. (c) NMI matrix for residues within Cluster 12 that indicate the dependencies between the pairs of amino acid positions. Residues of the classical Hallmark signature are indicated. (d) 3D‐alignment of six PDB structures (PDB codes 1BZQ, 1F2X, 1IEH, 1KXV, 1QD0, 1SJX) with different Hallmark signatures. Key residues are indicated in the same color coding as in Figure 1 and position 40 is indicated in yellow.

The VHH NGS data from Deszynski et al. (2022) holds extensive information regarding sequence variability, dependencies and relationships between amino acids at specific positions, potentially reflecting functional and structural relevance. To access this information and identify functionally relevant sequence patterns, we computed normalized Shannon entropy for each position in the multiple sequence alignment of the entire NGS dataset (Figure 8a), providing a measure of (un)certainty associated with each amino acid position. From this, we derived the normalized mutual information (NMI) matrix (see Section 5, Figure 9a and Table S1), which provides insights into the relationships and dependencies between all pairs of amino acid positions in the multiple sequence alignment: NMI values of 1 indicate complete dependency, that is, an amino acid in one position completely determines the amino acid in another position and vice versa, whereas a value of 0 indicates no dependency at all. Remarkably, the average values of NMI for the NGS dataset resulted in 0.02, meaning that on average only 2% of information is shared between pairs of amino acid locations. Hierarchical clustering was then performed using the NMI matrix to identify sequence patterns with high mutual information and dependency, thus revealing potential functional relationships within the NGS dataset of VHH sequences. Figure 9b shows the dendrogram associated with the hierarchical clustering and the corresponding rearranged NMI matrix.

The clustering of the NMI on the NGS dataset reveals distinct groupings of IMGT regions and their cluster memberships (see Table S2). Clusters 1, 2, and 3 predominantly include core positions associated with the CDR3 region, reflecting the expected high level of correlation in this region. Clusters 4 and 5 span CDR1 and both FR1 and FR3 regions, respectively. Clusters 6 and 7 are specific to CDR2 and CDR3 regions, with Cluster 7 also including sequences from FR4. Cluster 8 is the largest and most diverse, covering regions from FR1, FR2, FR3, and FR4. Smaller clusters like Cluster 9 and Cluster 10 combine FR and CDR regions. Cluster 11 is extensive, covering FR1, FR2, CDR2, FR3, and CDR3 regions. Cluster 16 is also diverse, spanning various IMGT regions such as FR1, FR2, FR3, and FR4, as well as CDR1 and CDR2. These clustering patterns aligns with the expectation that typical antibody regions, including FRs and CDRs, are mixed due to their functional interplay, mutual information, and interaction patterns.

Notably, Cluster 12 comprises the Hallmark positions 42, 49, 50, and 52, along with position 40 (assigned to FR2 according to IMGT, and CDR1 according to the Kabat numbering scheme), showing a significantly higher mutual dependency of 15%–37% compared to the 2% average for all pairs of positions (Figure 9c). This suggests a high degree of shared information among these residues, reflected in specific sequence motifs observed in the dataset. Frequency analysis over the entire NGS data set reveals that the most prevalent residues in this non‐Hallmark position 40 are Gly (63.9%), Ala (13.7%) or Ser (10.6%), see Table S3. However, analysis of logo plots for the different NGS sequence subsets (FERG, VGLW, YQRL, FERF; Figure 8a) reveals that the relative frequency of residues observed in position 40 is significantly different, depending on the present Hallmark motif: While Gly occurs most often in position 40 when the Hallmark motif is FERG, YQRL or FERF, the most prevalent residues in combination with the VGLW Hallmark signature are Ser (51.2%), Thr (14.1%), Tyr (10.9%) or Asn (10.0%). All these residues can form intramolecular or intramolecular hydrogen‐bonds via their sidechains. As evident from Figure 7, these different sequence patterns of residues 40, 42, 49, 50, and 52, are also encoded in llama and alpaca germline sequences, again suggesting structural and functional relevance of this sequence signature. Structural visualization of residue 40 indeed shows that it is part of a structural patch on FR2 (according to IMGT numbering) together with the other Hallmark residues (Figure 9d). Based on these observations, it is tempting to extend the Hallmark definition by residue 40, since it might contribute together with the classical Hallmark signature to either stabilize CDR3 in the bioactive conformation or to establish interactions with antigens.

## 3 DISCUSSION

By integrating experimental studies with in silico characterizations, this study presents an in‐depth investigation into the structural determinants governing the functional properties of VHHs, with a focus on the humanization and optimization of two llama‐derived VHHs targeting the natural cytotoxicity receptor NKp30. Only few studies, such as those by Soler et al. (2021) or Vincke et al. (2009), have delved into the effects of systematic mutations on Hallmark residues, including experimental structural analysis and investigations of binding affinity. Our exploration of mutations on Hallmark residues on VHH1 and VHH2 and their systematic analysis in terms of binding affinity against NKp30 in combination with experimental and in silico structure characterization, positions this study among the first to address the effects of Hallmark humanization in a prospective study comprehensively.

Furthermore, this study marks the first to report the crystal structure of NKp30 in complex with a single‐domain antibody, shedding light on the molecular interactions governing the activation of this receptor on NK cells and T cell subsets that can be exploited for effector cell redirection (Correia et al., 2018; Kellner et al., 2012; Pekar et al., 2021). The application of AlphaFold2 for the VHH1‐NKp30 complex allowed us to generate a structural hypothesis to rationalize the conformational fidelity of VHH1, aligning closely with the apo VHH1 X‐ray structure and demonstrating consistent agreement with previous epitope binning data: Both VHHs address different epitopes on NKp30, with VHH1 overlapping with the epitope of the natural ligand B7H6. Notably, the presence of rare Hallmark motifs FARS and VEHG in both VHH1 and VHH2, alongside the observation that partial humanization of these Hallmark motifs also functions effectively, underscores the complexity of VHH‐antigen interactions and the need for tailored approaches to VHH humanization. The roles of the Hallmark regions differ considerably between the two VHHs: In VHH2, the Hallmark region does not contribute to the paratope but appears crucial for constraining CDR3 into its bioactive conformation. Conversely, in VHH1, the Hallmark residues are—according to the AlphaFold2‐generated hypothesis—integral to the NKp30 paratope rather than directly interacting with or stabilizing CDR3. Interestingly, conformational stabilization of CDR3 is facilitated by the presence of the non‐canonical disulfide in VHH1.

MD simulations of the VHH variants in solution suggested that decreases in binding affinity resulting from specific Hallmark residue mutations, is accompanied by an increase in conformational diversity and consequently a higher conformational entropy. This increase in conformational diversity can be accompanied by a population shift resulting in multiple weakly populated states, reducing the probability of the binding competent conformation (Figure 6) (Fernández‐Quintero et al., 2021). The conformational entropy of proteins, manifested by the presence of metastable states that rapidly interconvert, not only plays a crucial role in protein folding and stability (Guarnera & Vanden‐Eijnden, 2016), but also in protein–protein and protein‐ligand binding interactions. Various binding processes are governed by enthalpic interactions, however, structural dynamics and heterogeneity contributing to conformational protein entropy are additional important determinants for molecular recognition and high affinity binding (Frederick et al., 2007; Löhr et al., 2022; Mikolajek et al., 2022; Seidler et al., 2023). Thus, the observed increase in entropy further highlights the important role of Hallmark residues and non‐canonical cysteine bridges in conformational preselection and determining the shape and dynamics of the paratope due to the strong structural correlation between Hallmark residues, Vernier zone residues and all CDRs, including CDR1 and CDR2 (Fernández‐Quintero et al., 2020; Gaudreault et al., 2022). This is also confirmed by studies showing that alterations in the FRs can induce conformational changes that affect the CDRs' orientation and dynamics, therefore influencing antigen binding affinity and specificity (Mitchell & Colwell, 2018).

Based on the distinct structural and functional roles of Hallmark motifs and the non‐canonical cysteine for these two specific VHHs, we scrutinized the functional significance of Hallmark motifs and non‐canonical cysteine bridges in VHHs in general. As complement to previous studies (Bahrami Dizicheh et al., 2023; Conrath et al., 2003; Deszyński et al., 2022; Gordon et al., 2023; Kuroda & Tsumoto, 2023; Li et al., 2016; Mitchell & Colwell, 2018; Murakami et al., 2022; Muyldermans et al., 1994; Nguyen et al., 2000), we conducted a systematic analysis of germline and NGS sequences, as well as PDB structures that revealed significant diversity in Hallmark motifs and a prevalent utilization of non‐canonical disulfide bonds. Alongside these publications, the results from our study underscore the essential role of Hallmark residues and non‐canonical disulfides in VHHs: In contrast to classical antibodies, wherein the VL chain increases paratope size and constrains the H‐CDR3 conformational space, VHHs exhibit heightened diversity in CDR3 length and conformation. Hallmark residues and non‐canonical disulfides emerge as key modulators of this diversity, serving to stabilize CDR3 conformation and expand sequence and structural paratope size and diversity.

### 3.1 Implications for VHH humanization and sequence optimization

A recent review (Gordon et al., 2024) has highlighted the challenge of applying in silico humanization protocols designed for conventional antibodies to VHHs due to their sequence and structural differences. Our study's results on VHH1 and VHH2 reaffirm previous findings, indicating that complete humanization of VHH FRs, including Hallmark residues, can compromise antigen binding or surface hydrophobicity. While this study illuminates the diverse mechanisms employed by VHHs to optimize their paratopes, it also underscores the indispensable role of complex structures, such as the herein reported NKp30‐VHH complexes, in rationalizing the structural effects observed in NKp30‐binding VHHs: The different effects of partial and full humanization in Hallmark or Vernier regions on VHH1 and VHH2 on binding affinity against NKp30 would not have been possible to rationalize in the absence of the complex structures. While we were able to obtain a high resolution x‐ray structure for the NKp30‐VHH2 complex, the NKp30‐VHH1 complex structure was modeled using AlphaFold2. Remarkably, the predicted bioactive conformation of VHH1 is very similar to its apo x‐ray structure (Cα rmsd: 0.66 Å), although this structure was not part of the AlphaFold2 training set. Furthermore, the AlphaFold2‐predicted NKp30‐VHH1 was useful to rationalize the available epitope binning and SAR data, in particular the affinity loss of the G52W mutation. However, we would like to emphasis that this agreement with experimental data does not explicitly prove a high accuracy for this modeled complex and for AlphaFold2's accuracy in predicting antibody–antigen complexes in general: Although great progress has been achieved in the in silico prediction of antibody–antigen complexes with AlphaFold2, the success rate in identifying near–native complex poses as the top hit is currently only about 30–50% (Yin & Pierce, 2024). Indeed, while the AlpaFold2 generated VHH1‐NKp30 complex rationalizes the available experimental epitope binning and SAR data, an AlphaFold2‐generated binding hypothesis of the VHH2‐NKp30 complex does not agree with our X‐ray structure (Supplementary Figure S2).

However, due to the time‐consuming process of protein production and crystallization, experimental high‐resolution VHH‐antigen complex structures are usually not available before humanization and sequence optimization. Therefore, the ultimate question is: how can we pragmatically humanize and sequence‐optimize VHH sequences in one DMTA cycle with as little design variants as possible in the absence of VHH‐antigen complex structures? One important, but often overlooked consideration in VHH humanization is the definition of CDRs and FRs. As depicted in Figure 1, the CDRs in the Kabat, Chothia, and IMGT systems encompass overlapping, yet not identical sequence segments, owing to their distinct criteria (Dondelinger et al., 2018). VHH humanization of the FR will result in different sequences based on the applied numbering scheme. As evident (i) from the logo plot (Figure 9a), (ii) from previous structural studies about VHH‐antigen contact preferences (Mitchell & Colwell, 2018) and (iii) from the germline repertoire sequence alignment (Figure 7), residues on the fringes of CDRs (according to individual numbering schemes) are structurally and functionally relevant for the overall bioactive conformation of VHHs and their humanization can consequently lead to reduced functionality. Within our study on VHH1 and VHH2 humanization, we defined CDR1‐3 as those regions that comprise a combined IMGT‐Kabat‐Chothia definition. As a result, residue 40, which was suggested from this study to be mutually dependent from the Hallmark residues, would be within FR2 according to the IMGT definition, but is within CDR1 according to Kabat and the combined IMGT‐Kabat‐Chothia definition and was therefore not humanized in VHH1 and VHH2. Indeed, structural analysis of the VHH1 and VHH2 structures suggests that humanization of residue 40 (G40S in both cases) would result in alteration of the CDR3 conformation, potentially impacting binding against NKp30 (see Figure S3). Similarly, the non‐canonical Cys55 is located in FR2 according to the IMGT and Chothia definition, but in CDR2 according to the Kabat and the combined IMGT‐Kabat‐Chothia definition (Figure 1). Therefore, framework‐based humanization with the combined IMGT‐Kabat‐Chothia CDR definition is expected to reduce the risk to destabilize the bioactive paratope conformation.

Building on previous publications and our current study, we recommend employing a framework‐based humanization approach using the combined IMGT‐Kabat‐Chothia CDR definition. Additionally, we propose a generic risk scoring scheme for humanizing specific positions, building on Sulea's previous suggestions (Sulea, 2022). The proposed risk assessment entails:

- Highest risk for humanization of Hallmark positions 42 and 52, crucial for antigen binding or bioactive conformation stabilization.
- High risk for humanizing non‐canonical cysteine residues involved in disulfide bridges with CDR3, essential for maintaining the bioactive conformation.
- High risk for humanizing Hallmark residues 49 and 50 to increase hydrophobicity and reduce solubility.
- Significant risk for humanizing Vernier zone residues, potentially impacting the bioactive conformation.
- Moderate risk for humanization of additional residues within IMGT positions 80–87 (DE loop).

Another important consideration in VHH humanization is the extent to which it is necessary to achieve a maximal degree of human‐likeness. An increased overall human‐likeness of antibodies does not per se guarantee reduced immunogenicity, since no strict linear correlation with reported percent ADAs in clinical trials can be established (Martin et al., 2023). Immunogenicity is known to be triggered by further various factors beyond human‐likeness, such as antibody aggregation (Lundahl et al., n.d.), chemical degradation (Hermeling et al., 2004), or non‐germline peptide stretches occurring at the CDR/FR junctions (Gordon et al., 2024; Sulea, 2022). Consequently, it is more pragmatic to consider not just human‐likeness (Sang et al., 2022), but also an overall beneficial (in silico) developability profile in sequence humanization and optimization. This includes orthogonal factors such as physical and chemical stability and a proxy metric for the likelihood of peptide fragments along the sequence that reflect a “human nativeness” score (Prihoda et al., 2022; Ramon et al., 2024) to reduce the risk/likelihood for MHC‐II binding, as detailed in (Gordon et al., 2024).

## 4 CONCLUSION

In conclusion and in agreement with prior recommendations (Sulea, 2022; Vincke et al., 2009), we advocate commencing humanization endeavors from a ‘low risk variant’ (v1.2 in Figure 1). This involves humanizing the camelid VHH sequence in non‐critical structural positions, specifically those outside the CDRs (using the combined IMGT‐Kabat‐Chothia definition) and outside Hallmark, Vernier zone and non‐canonical cysteine positions. Based on analysis of literature reports and internal VHH humanization campaigns, such a ‘low risk variant’ typically maintains a binding affinity similar to the parental sequence, unless VHH‐antigen binding occurs via atypical binding modes. (Braun et al., 2016) Further design variants should be derived from this ‘low risk variant’ and include the diversification of Vernier zone residues in the FR and—with a lower priority—Hallmark positions. Given that additional mutations may be necessary to optimize the overall early developability profile, the number of variants that can be produced and tested experimentally strongly depend on the overall complexity of the mutational landscape involved. To finalize on a sequence that is sufficiently optimized after one DMTA cycle, it would be preferable to express and test all combinatorial variants at once, however this may exceed the available capacity if multiple residues are diversified in all combinations. Therefore, we recommend ranking and filtering mutations based on a generic risk scoring scheme as outlined above, and relevant in silico property predictions, or to even frontload experimental high‐throughput assays on the parental VHH sequence, such as accelerated forced chemical degradation tests, to further prioritize design variants in functionally relevant positions. Looking ahead, it is worth to emphasize that the humanization protocol described in this article can be fully automated. By integrating computational tools and machine learning algorithms, we can streamline the identification and modification of target residues, thereby accelerating the development of humanized VHH variants with improved developability profiles. This approach will enhance efficiency and reproducibility, paving the way for more rapid advancements in VHH therapeutic development.

## 5 MATERIALS AND METHODS

### 5.1 Protein expression and analysis

For the present study, variants of VHH1 and VHH2 were synthesized as bispecific VHH SEEDbodies as in our previous study (Klausz et al., 2022). The SEED (strand‐exchanged engineered domains) technology relies on complementary beta‐strand exchanges within IgG and IgA CH3 domains, resulting primarily in heavy chain heterodimers. VHH variants were cloned into a pTT5 mammalian expression vector (Thermo Fisher Scientific) as N‐terminal fusion to the hinge region of Fc SEED AG chains, consequently enabling the production of bispecific SEEDbodies in combination with humanized cetuximab Fab (hu225) fused to the SEED GA chain. All proteins were expressed in the ExpiHEK293F Expression System (Thermo Fisher Scientific), in 25 ml scale according to the manufacturer's manual standard protocol with 2:2:1 ratio of light chain to AG to GA chain. After 7 days, the protein containing supernatants were purified with MabSelect antibody purification chromatography resin (Cytiva). After sterile filtration, protein concentrations were determined by A280 absorption measurement. For the assessment of protein sample quality regarding monomer content [%], analytical SEC was applied using 7.5 μg protein per sample on a TSKgel UP‐SW3000 column (2 μm, 4.6 × 300 mm, Tosoh Bioscience) on an Agilent high performance liquid chromatography (HPLC) 1260 Infinity system with a flow rate of 0.35 ml/min using 50 mM sodium phosphate, 0.4 M NaClO4 pH 6.3 as mobile phase. Signals were recorded at 214 nm.

### 5.2 Biolayer interferometry (BLI)

Binding properties of the evaluated molecules were assessed using an Octet Red BLI system (Sartorius). Binding experiments were conducted in KB buffer (PBS pH 7.4, 0.1% BSA, 0.02% Tween‐20) using Anti‐Human Fc Capture (AHC) biosensors. For qualitative binding assessment, biosensors were loaded with the bispecific protein samples at a concentration of 10 μg/ml for 120 s before 60 s of sensor rinsing in KB and consecutive association of rh NKp30 antigen at 100 nM for 180 s before final dissociation step in KB buffer for another 180 s. To quantify binding behavior of the positive binders, the samples were immobilized at 5 μg/ml for 180 s on AHC biosensors and subjected to a twofold serial dilution of recombinant human (rh) NKp30, starting at a concentration of 100 nM using a measurement window of 300 s for association and 600 s for dissociation. The data were aligned to the association step, and inter‐step correction was applied at the dissociation step, as well as noise reduction using Savitzky–Golay filtering. The resulting data were analyzed using a 1:1 binding model. Fitted BLI traces are shown in Figure S4.

### 5.3 Cloning, expression, and purification of human NKp30, VHH1 and VHH2

The gene encoding for human NKp30 truncated variant (residues 19–130) UniProtKB O14931 was cloned into an expression pTT5 (Durocher et al., 2002) vector with CEACAM5 signal peptide (1–34) using EcoRI and BamHI restriction enzymes in frame with a C‐terminal HRV‐3C protease cleavage site and an His8 Tag (Genscript, Rijswijk, Netherlands). Suspension human embryonic kidney derived cell lines: HEK293‐6E cells (Durocher et al., 2002) were cultured in 5 L shake flasks (Corning) containing FreeStyle F17 medium, supplemented with 4 mM GlutaMax, and 0.1% (v/v) Pluronic F‐68 (Gibco). HEK293‐6E cells were transfected with 1 mg of DNA pTT5_NKp30 (CEACAM_SP 1–34; 19–130)‐3C‐His construct/L of culture, with polyethylenimine (PEI) on a proportion of 2:1 (PEI:DNA) at a cell density of approximately 2.2 × 10 (Boje et al., 2024) cell/ml (Polysciences, Inc). Cell density and cell viability were determined using Vi‐cell BLU (BECKMAN COULTER). Cells were kept at 37°C, 90 rpm (Infors HT Multitron incubator), in a humidified atmosphere with 5% CO_2_ for 5 days. Conditioned media was collected by centrifugation (11,295×*g*, 10 min at 4°C) and was loaded in 2x HisTrap excel 5 ml column. Unspecific proteins were removed by washing the resin with PBS 1× pH 7.4. The protein of interest was eluted between 90 mM and 450 mM Imidazole. Eluted fractions were analyzed by SDS‐PAGE and Anti‐His Western Blot (monoclonal Anti‐polyHistidine antibody—H1029, Sigma‐Aldrich). Selected fractions were pooled together, HRV‐3C‐His protease was added, and the mixture was inserted into a dialysis membrane, and left overnight at 4°C in dialysis buffer (PBS 1x pH 7.4) to reduce Imidazole concentration. The sample was re‐injected into a HisTrap HP column pre‐equilibrated with PBS 1x pH 7.4, 20 mM Imidazole. The protein of interest was collected from the flow‐through. The NKp30 selected fraction was concentrated and applied to a Superdex 75 26/60 column equilibrated with PBS 1x pH 7.4. Selected fractions were polled together and concentrated to 21.5 mg/ml (concentration determined by BCA method).

The VHH fragments of VHH1 and VHH2 for crystallization were expressed as C‐terminally polyhistidine‐tagged fusions in ExpiHEK293F™ cells and purified via immobilized metal ion affinity chromatography.

### 5.4 Human NKp30 complex preparation with VHH2

Human NKp30 was incubated with VHH2 in a molar ration of 1.05:1, overnight at 4°C. The complex was purified in a Superdex 75 16/60 column equilibrated with 25 mM Tris–HCl pH 7.5, 150 mM NaCl. Selected fractions were pooled together and concentrated to 32.1 mg/mL.

### 5.5 NKp30:VHH2 complex crystallization

NKp30:VHH2 complex crystals were obtained at 20°C in a crystallization condition containing 20% (v/v) PEG 4000, 20% (v/v) Isopropanol, 0.1 M Sodium citrate pH 5.6 with microseeding, using the sitting drop vapor diffusion method, with the drop consisting of 100 nL of protein complex mixed with 70 nL of precipitant solution and 30 nL of seeds. VHH1 *apo* (36.8 mg/mL) was crystallized at 20°C in a condition composed of 1.5 M Ammonium phosphate, 0.1 M Sodium citrate pH 5.5, using the sitting drop vapor diffusion method, with the drop consisting of 100 nL of protein complex, 70 nL of precipitant solution and 30 nl of seeds.

### 5.6 Crystallographic data acquisition, processing, and structure solution and refinement

NKp30:VHH2 complex crystals were harvested and cryopreserved for X‐ray diffraction by transfer to a solution consisting of 20% (v/v) PEG 4000, 20% (v/v) Isopropanol, 0.1 M Sodium citrate pH 5.6, 5% (v/v) glycerol. VHH1 *apo* crystals were harvested and cryopreserved in a solution of 2 M Ammonium phosphate, 0.1 M Sodium citrate pH 5.5, 20% (v/v) PEG 400. X‐ray diffraction data for both NKp30:VHH2 complex and VHH1 *apo* were collected on the X10SA (PXII) beamline at the Swiss Light Source (SLS) synchrotron radiation source using a EIGER2 16 M (up to 550 Hz) detector. Diffraction image data reduction was performed using the program autoPROC. NKp30:VHH2 complex structure was determined by Molecular replacement using PHASER (McCoy et al., 2007), and refined using Buster. Data collection, processing and refinement statistics can be found in Tables S4 and S5.

### 5.7 MD simulations

As starting structures for our simulations, we used the available X‐ray structures of VHH1 (PDB code 9FXF) and VHH2 (PDB code 9FWW). For the selected variants, we performed homology model generation and preparation for the MD simulations in MOE (Molecular Operating Environment, 2022.02. Chemical Computing Group ULC, Montreal, QC, Canada) using the respective X‐ray structure as template. We adapted a previously published simulation protocol to characterize the CDR loop diversity in solution and to structurally elucidate the influence of specific residues on the paratope (Fernández‐Quintero et al., 2019; Fernández‐Quintero, Fischer, et al., 2022; Seidler et al., 2023).

To broaden the exploration of conformational space, we employed well‐tempered bias‐exchange metadynamics simulations within GROMACS, utilizing the PLUMED 2 software (Barducci et al., 2008; Bauer et al., 2022; Bonomi et al., 2009; Bonomi et al., 2019; Domene et al., 2015; Tribello et al., 2014). As collective variables we chose a previously established protocol, boosting a linear combination of sine and cosine functions of the ψ torsion angles of the CDR loops and the DE loop (Fernández‐Quintero, DeRose, et al., 2022; Fernández‐Quintero, Fischer, et al., 2022). The collective variables were computed using PLUMED 2 functions MATHEVAL and COMBINE (Tribello et al., 2014). As previously discussed, the ψ torsion angle, is sufficient to capture conformational transitions comprehensively (Ramachandran et al., 1963). Additionally, we incorporated random exchanges to facilitate interchange among replicas.

For our simulations, we employed a Gaussian height of 5 kJ/mol, with Gaussian deposition occurring every 5000 steps and a bias factor of 10. We performed 500 ns of bias‐exchange metadynamics simulations for each of the investigated nanobody variants. The obtained trajectories were aligned on the VHH and clustered on the CDR loops using the average linkage hierarchical clustering algorithm with an RMSD cut‐off criterion of 1.2 Å implemented in cpptraj, resulting in a large number of clusters. The respective cluster representatives were then equilibrated and used as starting structures for each 100 ns of classical molecular dynamics simulations using the AMBER 22 simulation package (Case et al., 2023). MD simulations were performed in an NpT ensemble using pmemd.cuda (Salomon‐Ferrer et al., 2013). Bonds involving hydrogen atoms were restrained by applying the SHAKE algorithm, allowing a time step of 2 fs. Atmospheric pressure of the system was preserved by weak coupling to an external bath using the Berendsen algorithm (Berendsen et al., 1984). The Langevin thermostat was used to maintain the temperature during simulations at 300 K (Adelman & Doll, 1976; Doll et al., 1975). With the obtained trajectories, we performed a time‐lagged independent component analysis (tICA) via the Python library PyEMMA 2 using the backbone torsions of the respective CDR loops as input features, employing a lag time of 25 ns (Scherer et al., 2015). tICA allows to identify the slowest motions within the systems, facilitating a kinetic discretization of the sampled conformational space (Pérez‐Hernández et al., 2013; Schwantes & Pande, 2013). This dimensionality reduction technique detects slow‐relaxing degrees of freedom, facilitating kinetic clustering, a prerequisite for constructing a Markov‐state model (MSM). Thermodynamics and kinetics were reconstructed based on the respective tICA conformational spaces using an MSM, also implemented in PyEMMA 2. This involved employing the k‐means clustering algorithm to define microstates and the PCCA+ clustering algorithm to coarse‐grain these microstates into macrostates (Ikotun et al., 2023; Röblitz & Weber, 2013). MSMs, as network models, offer insights into conformational states and transition probabilities. Basically, MSM's provide a coarse‐grained view of the system, reflecting the free energy surface and ultimately determine the structure and dynamics of the system. Thereby, MSMs provide a unique understanding of states and transition probabilities, facilitating the comparison with experimental data (Prinz, Wu, et al., 2011). Sampling efficiency and MSM reliability, including optimal feature mappings, were assessed using the Chapman‐Kolmogorov test (see Figure S5 for variants of VHH2 shown in Figure 6), employing the variational approach for Markov processes and monitoring the fraction of states used (Prinz, Keller, & Noé, 2011; Prinz, Wu, et al., 2011). Fully connected network states are required to estimate transition kinetics and relative equilibrium probabilities. For building the MSM, backbone torsions of the CDR‐H3/CDR‐H2, all CDR loops were considered. We defined 100–150 microstates using the k‐means clustering algorithm, applying a lag time of 25 ns. Cluster analyses for specific loops were performed in cpptraj using the implemented average linkage clustering, as well as the B‐factor (Roe & Cheatham, 2013).

### 5.8 VHH NGS data

NGS VHH sequences were downloaded from https://research.naturalantibody.com/nbdownload and all sequences were annotated and aligned using ANARCI (Dunbar & Deane, 2016). Sequences that were not fully annotated were removed from the dataset, resulting in 9,869,030 sequences that were used for the analyses within this study.

### 5.9 Calculation of Shannon entropy, normalized mutual information and hierarchical clustering

To assess the information content and variability within the NGS dataset, we first calculated the Shannon entropy for each position in the multiple sequence alignment. The Shannon entropy $H\left(X\right)$ for a position 𝑋 is defined as:

$$H\left(X\right)=-\sum_{i}p\left(x_{i}\right)\log p\left(x_{i}\right)$$

where $p\left(x_{i}\right)$ is the probability of the amino acid type $x_{i}$ at position 𝑋. A normalized version of the Shannon entropy reads:

$$\mathrm{NH}\left(X\right)=\frac{H\left(X\right)}{\log21}$$

where the log21 denominator is the upper limit of the Shannon entropy for a complete set of 20 amino acid type and one gap. In this way, the $\mathrm{NH}\left(X\right)$ metric quantifies the degree of uncertainty associated with each amino acid position in a range between 0 and 1, where $\mathrm{NH}\left(X\right)=0$ is the case of lowest uncertainty – one amino acid type with 100% probability at location *X* – and $\mathrm{NH}\left(X\right)=1$ corresponding to the case of highest uncertainty, that is, all amino acids have same probability at location *X*. As a next step, the mutual information (MI) was computed to evaluate the shared information between pairs of positions:

$$\mathrm{MI}\left(X,Y\right)=\sum_{i,j}p\left(x_{i},y_{j}\right)\log\frac{p\left(x_{i},y_{j}\right)}{p\left(x_{i}\right)\;p\left(y_{j}\right)}$$

where $p\left(x_{i},y_{j}\right)$ is the joint probability distribution of the pair of amino acid positions *X* and 𝑌, and $p\left(x_{i}\right)$ and $p\left(y_{j}\right)$ are the marginal probabilities of *X* and *Y*, respectively. MI measures the amount of information shared between to amino acid positions. To normalize the mutual information and account for differences in entropy between positions, we calculated the normalized mutual information (NMI) between positions 𝑋 and 𝑌 as:

$$\mathrm{NMI}\left(X,Y\right)=\frac{2\;\mathrm{MI}\left(X;Y\right)\;}{H\left(X\right)+H\left(Y\right)}$$

This normalization ensures that the MI values are scaled between 0 and 1, allowing for a direct comparison of dependencies across different pairs of amino acid positions. A $\mathrm{NMI}\left(X,Y\right)$ value of 1 indicates complete dependency between the locations *X* and *Y*, whereas a value of 0 indicates no dependency at all. In other words, complete dependency between locations *X* and *Y* implies that the amino acid type in position *X* completely determines the amino acid type in *Y* and vice versa. Using a hierarchical clustering and Ward's linkage method, clusters of amino acid locations were identified (chosen cutoff *d* = 1.1) with the aim to maximize the NMI values between pairs of members within each cluster.

### 5.10 VHH PDB structures

VHH PDB structures were extracted using the Antibody Protein Family Database in the molecular modeling software package MOE (2020.09: Chemical Computing Group Inc.; 2020). Visualization of 3D structures was done with PyMOL (The PyMOL Molecular Graphics System, Version 2.0 Schrödinger, LLC.).

### 5.11 Sequence annotation and in silico sequence developability assessment using SUMO

Our internal pipeline termed “Sequence Assessment Using Multiple Optimization Parameters (SUMO)” (Evers et al., 2023) automatically annotates VHH sequences according to different numbering schemes (including IMGT, Kabat and Chothia), builds structural models based on the provided sequences of the variable regions, calculates human‐likeness by sequence comparison to the most similar human germline sequence and identifies structure‐based surface‐exposed chemical liability motifs (unpaired cysteines, methionines, asparagine deamidation motifs and aspartate deamidation sites) as well as sites susceptible to post‐translational modification (N‐linked glycosylation). Moreover, a small set of orthogonal computed physico‐chemical descriptors are calculated for the complete variable domain as well as the CDRs. This combined information is used as starting point for sequence humanization and further optimization. Importantly, our CDR1‐3 definition comprises all residues that are assigned as CDR by the IMGT, Kabat or Chothia CDR definition.

The data that supports the findings of this study are available in the supplementary material of this article.

## AUTHOR CONTRIBUTIONS

**Monica L. Fernández‐Quintero:** Investigation; writing – original draft; visualization; methodology; funding acquisition. **Enrico Guarnera:** Conceptualization; methodology; software; visualization; writing – review and editing. **Djordje Musil:** Investigation; formal analysis; writing – review and editing; data curation; writing – original draft. **Lukas Pekar:** Investigation; formal analysis; data curation; writing – review and editing. **Carolin Sellmann:** Formal analysis; writing – review and editing; data curation. **Filipe Freire:** Investigation; data curation; formal analysis; writing – review and editing. **Raquel L. Sousa:** Investigation. **Sandra P. Santos:** Investigation. **Micael C. Freitas:** Investigation. **Tiago M. Bandeiras:** Investigation. **Margarida M. S. Silva:** Investigation. **Johannes R. Loeffler:** Investigation; writing – review and editing. **Andrew B. Ward:** Supervision; writing – review and editing; funding acquisition. **Julia Harwardt:** Data curation; formal analysis; writing – review and editing. **Stefan Zielonka:** Conceptualization; data curation; investigation; validation; supervision; resources; project administration; writing – original draft; writing – review and editing. **Andreas Evers:** Conceptualization; methodology; software; data curation; investigation; validation; supervision; visualization; project administration; writing – original draft; writing – review and editing.

## CONFLICT OF INTEREST STATEMENT

Lukas Pekar, Andreas Evers and Stefan Zielonka filed a patent application based on sequences and experimental data from this study. In addition, Andreas Evers, Enrico Guarnera, Djordje Musil, Lukas Pekar, Carolin Sellmann, Julia Harwardt and Stefan Zielonka are employees at Merck Healthcare KGaA. Besides, this study was conducted in the absence of any further commercial interest.

## Supporting information


**DATA S1.** Supporting Information.

**TABLE S1.** Normalized mutual information (NMI) matrix based on normalized Shannon entropies along the VHH sequence (in IMGT numbering).
