# Supplementary material for: On the humanization of VHHs: Prospective case studies, experimental and computational characterization of structural determinants for functionality
Source: Protein Sci. 2024 Oct 18;33(11):e5176. doi: 10.1002/pro.5176 (PMC11487682; doi:10.1002/pro.5176)
Supplement: Supplementary file 1 — DATA S1. Supporting Information. [file PRO-33-e5176-s001.docx]

**Supplementary Information:**

**Title: "On the humanization of VHHs: Prospective case studies, experimental and computational characterization of structural determinants for functionality"**

Monica L. Fernández-Quintero^1^, Enrico Guarnera^2^, Djordje Musil^3^, Lukas Pekar^2^, Carolin Sellmann^2^, Filipe Freire^4^, Raquel L. Sousa^4^, Sandra P. Santos^4^, Micael C. Freitas^4^, Tiago M. Bandeiras^4^, Margarida M. S. Silva^4^, Johannes R. Loeffler^1^, Andrew B. Ward^1^, Julia Harwardt^2^, Stefan Zielonka^2,5^, Andreas Evers^2^

^1^Department of Integrative Structural and Computational Biology, The Scripps Research Institute, La Jolla, CA 92037, USA

^2^Antibody Discovery and Protein Engineering, Merck Healthcare KGaA, Darmstadt, Germany

^3^Structural Biology and Biophysics, Merck Healthcare KGaA, Darmstadt, Germany

^4^iBET, Instituto de Biologia Experimental e Tecnológica, Apartado 12, 2781-901 Oeiras, Portugal.

^5^Institute for Organic Chemistry and Biochemistry, Technical University of Darmstadt, Darmstadt, Germany

Corresponding authors:

Andreas Evers, Antibody Discovery & Protein Engineering, Merck Healthcare KGaA, Frankfurter Straße 250, D-64293 Darmstadt, Germany, E-mail: [Andreas.Evers@merckgroup.com](mailto:Andreas.Evers@merckgroup.com). <https://orcid.org/0000-0003-4643-1941>; and Monica L. Fernández-Quintero, https://orcid.org/0000-0002-6811-6283, E-mail: [mfernandez@scripps.edu](mailto:mfernandez@scripps.edu).

SupplementalTable1.xlsx

**Supplementary Table 1**. Normalized mutual information (NMI) matrix based on normalized Shannon entropies along the VHH sequence (in IMGT numbering).

**Supplementary Table 2**. Cluster memberships of sequence positions and their regions derived from the NMI matrix on the VHH NGS dataset.

| Clustering of the Normalized Mutual Information on NGS dataset (d=1.1) | |
| --- | --- |
| Cluster ID | **IMGT regions and cluster memberships** |
| 1 | **CDR3**: 111D, 111E, 111F, 112E, 112F, 112G |
| 2 | **CDR3**: 111B, 111C, 112C, 112D |
| 3 | **CDR3**: 110, 111, 111A, 112, 112A, 112B |
| 4 | **CDR1**: 31, 34 |
| 5 | **FR1**: 9, 16, 18; **FR3**: 76 |
| 6 | **CDR2**: 60, 61 |
| 7 | **CDR3**: 107, 108, 109, 113, 114, 115, 116, 117; **FR4**: 120 |
| 8 | **FR1**: 3, 4, 5, 6, 8, 13, 23; **FR2**: 41, 43; **FR3**: 70, 75, 79, 81, 89, 94, 98, 99, 102, 104; **FR4**: 119, 121, 124, 125, 126 |
| 9 | **FR1**: 19, 22; **FR3**: 77; **FR4**: 122, 127; |
| 10 | **FR1**: 24, 25, 26; **CDR1**: 27; **FR3**: 67 |
| 11 | **FR1**: 7, 11, 14, 17, 20, 21; **FR2**: 44, 45, 46, 47, 48, 51, 53; **CDR2**: 56, 65; **FR3**: 68, 69, 71, 72, 74, 78, 82, 83, 84, 85, 86, 87, 88, 90, 91, 92, 93, 95, 96, 97, 100, 103; **CDR3**: 105, 106, 118; |
| 12 | **FR2**: 40, **FR2+HM**: 42, 49, 50, 52; |
| 13 | **FR1**: 1, 2; **FR4**: 123, 128; |
| 14 | **FR1**: 12; **CDR1**: 38; **FR3**: 80, 101; |
| 15 | **CDR1**: 30, 35; **FR2**: 39, 55; |
| 16 | **FR1**: 15; **CDR1**: 28, 29, 36, 37; **FR2**: 54; **CDR2**: 57, 58, 59, 62, 63, 64; **FR3**: 66; |

**Supplementary Table 3**. Relative frequency of position 40 (IMGT numbering) residues over the entire (ALL) VHH NGS dataset and over different subsets.

|  | Residue **40** frequency distribution over different datasets | | | | | |
| --- | --- | --- | --- | --- | --- | --- |
| amino acid | ALL | Cys | FERG | VGLW | YQRL | FERF |
| **A** | 13.7 | 17.1 | 17.0 | 3.0 | 9.6 | 16.2 |
| **C** | 0.4 | 0.1 | 0.6 | 0.2 | 0.1 | 0.2 |
| **D** | 0.6 | 0.4 | 0.2 | 0.5 | 1.7 | 0.4 |
| **E** | 0.1 | 0.1 | 0.1 | 0.0 | 0.4 | 0.0 |
| **F** | 0.1 | 0.1 | 0.0 | 0.5 | 0.0 | 0.0 |
| **G** | 63.9 | 75.9 | 77.7 | 4.0 | 84.7 | 72.8 |
| **H** | 0.4 | 0.2 | 0.2 | 2.0 | 0.2 | 0.1 |
| **I** | 0.3 | 0.1 | 0.1 | 1.2 | 0.0 | 0.0 |
| **K** | 0.2 | 0.1 | 0.1 | 0.5 | 0.2 | 0.0 |
| **L** | 0.1 | 0.1 | 0.1 | 0.1 | 0.0 | 0.0 |
| **M** | 0.1 | 0.1 | 0.0 | 0.4 | 0.0 | 0.0 |
| **N** | 2.1 | 0.6 | 0.3 | 10.0 | 0.8 | 0.2 |
| **P** | 0.1 | 0.0 | 0.0 | 0.1 | 0.0 | 0.0 |
| **Q** | 0.1 | 0.1 | 0.0 | 0.0 | 0.0 | 0.0 |
| **R** | 1.8 | 0.3 | 0.2 | 0.8 | 0.5 | 0.5 |
| **S** | 10.6 | 2.3 | 1.6 | 51.2 | 1.3 | 8.6 |
| **T** | 3.3 | 1.3 | 0.9 | 14.1 | 0.2 | 0.6 |
| **V** | 0.6 | 0.8 | 0.8 | 0.2 | 0.2 | 0.3 |
| **W** | 0.0 | 0.0 | 0.0 | 0.1 | 0.0 | 0.0 |
| **Y** | 1.6 | 0.2 | 0.1 | 10.9 | 0.2 | 0.1 |

**Supplementary Table 4.** X-ray Data collection and processing statistics.

|  | NKp30-VHH2  PDB: 9FWW | VHH1  PDB: 9FXF | | | | | | | |  |  |  |  |  |
| --- | --- | --- | --- | --- | --- | --- | --- | --- | --- | --- | --- | --- | --- | --- |
| X-ray Source | PXII/X10SA (SLS^1^) | PX14 (DESY^2^) | | | | | | | |  |  |  |  |  |
| Wavelength [Å] | 1.00001 | 0.97626 | | | | | | | |  |  |  |  |  |
| Detector | Eiger2 16M | Pilatus 6M | | | | | | | |  |  |  |  |  |
| Temperature [K] | 100 | 100 | | | | | | | |  |  |  |  |  |
| Space Group | I422 | C2 | | | | | | | |  |  |  |  |  |
| Cell: a; b; c; [Å] | 156.46 156.46 89.36 | 88.02; 34.95; 46.27 | | | | | | | |  |  |  |  |  |
| α; β; γ; [°] | 90.0; 90.0; 90.0 | 90.0; 108.9; 90.0 | | | | | | | |  |  |  |  |  |
| Resolution [Å] | 1.84 (2.05-1.84)^3^ | 1.07 (1.17-1.07) | | | | | | | |  |  |  |  |  |
| Unique reflections | 29007 | 37662 | | | | | | | |  |  |  |  |  |
| Multiplicity | 13.5 (13.3) | 6.6 (4.8) | | | | | | | |  |  |  |  |  |
| Completeness^4^ [%] | 91.3 (73.0) | 75.9 (27.5) | | | | | | | |  |  |  |  |  |
| Rmeas ^5^ | 0.117 (1.694) | 0.073 (0.692) | | | | | | | |  |  |  |  |  |
| Mean(I)/σ(I) | 15.4 (2.0) | 12.4 (2.2) | | | | | | | |  |  |  |  |  |
| ^1^ SWISS LIGHT SOURCE (SLS, Villigen, Switzerland)  ^2^ Deutsches Elektronen-Synchrotron (Hamburg, Germany) | | | | | |  | | |  |  |  |  |  |  |
| ^3^ Values in parenthesis refer to the highest resolution  ^4^ $Ellipsoidal completeness$ as calculated by STARANISO option in autoPROC | | | | |  |  |  |  |  |  |  |  |  |  |
| ^5^$Rmeas=\frac{\sum_{h} \sqrt{\frac{n_{h}}{n_{h}-1}}\sum_{i}^{n_{h}} \left\vert\text{Î}_{h}-I_{h,i} \right\vert}{\sum_{h} \sum_{i}^{n_{h}} I_{h,i}}$ with $\text{Î}_{h}=\frac{1}{n_{h}}\sum_{i}^{n_{h}} I_{h,i}$ where $I_{h,i}$ is the intensity value of the *i*th measurement of *h* | | | | | |  | |  | | |  |  |  |  |
|  | | |  | | | |  | | | | |  |  |  |
|  | | | |  | | |  | | | | |  |  |  |
|  | | | |  | | |  | | | | |  |  |  |

**Supplementary Table 5.** X-ray Refinement Statistics.

|  | NKp30-VHH2  PDB: 9FWW | | VHH1  PDB: 9FXF | |  |  |  |  |  |
| --- | --- | --- | --- | --- | --- | --- | --- | --- | --- |
| Resolution [Å] | 77.59-1.84 | 24.52-1.07 | | |  |  |  |  |  |
| Number of reflections (working / test) | 29007/1521 | 37662/1942 | | |  |  |  |  |  |
| Rcryst [%] | 0.199 | 0.193 | | |  |  |  |  |  |
| Rfree [%]^1^ | 0.208 | 0.206 | | |  |  |  |  |  |
| Total number of atoms: |  |  | | |  |  |  |  |  |
| Protein | 1802 | 915 | | |  |  |  |  |  |
| Heteroatoms | 38 | 19 | | |  |  |  |  |  |
| Water | 208 | 104 | | |  |  |  |  |  |
| Deviation from ideal geometry:^2^ |  |  | | |  |  |  |  |  |
| Bond lengths [Å] | 0.008 | 0.016 | | |  |  |  |  |  |
| Bond angles [°] | 0.98 | 1.30 | | |  |  |  |  |  |
| Ramachandran plot: |  |  | | |  |  |  |  |  |
| Most favored regions [%] | 9138 | 93.0 | | | |  |  |  |  |
| Additional allowed regions [%] | 8.7 | 7.0 | | | |  |  |  |  |
| Disallowed regions [%] | 0.0 | 0.0 | | | |  |  |  |  |
| ^1^ Test-set contains 5% of measured reflections | |  | |  |  |  |  |  |  |
| ^2^ Root mean square deviations from geometric target values | |  | |  |  |  |  |  |  |
|  | |  | |  | | |  |  |  |
|  | |  | |  | | |  |  |  |
|  | |  | |  | | |  |  |  |

**Supplementary Figure 1**. In silico developability assessment of VHHs, including their sequence identity compared to the most similar human germline (MOST SIMILAR GERMLINE) either based on the entire variable chain region (SEQ-ID) or the framework region only (SEQ-ID FR), as well as for their total number of specific chemical liabilities and PTMs, i.e., non-canonical cysteines, methionine oxidations, asparagine deamidations or aspartate isomerizations, and N-glycosylations, in structurally exposed CDR residues as derived from automatically generated models. As calculated physicochemical developability descriptors (IN SILICO PHYSCHEM), structure-based pI values (pIfv 3D), hydropbobic surface patches of the entire variable regions, and the CDR regions only, as well as positive patches of the CDRs. The complementing color coding indicates scores within one standard deviation from a benchmark mean (dataset of 77 biotherapeutics approved for human application) as green, scores above one standard deviation as yellow, and scores above two standard deviations as red.


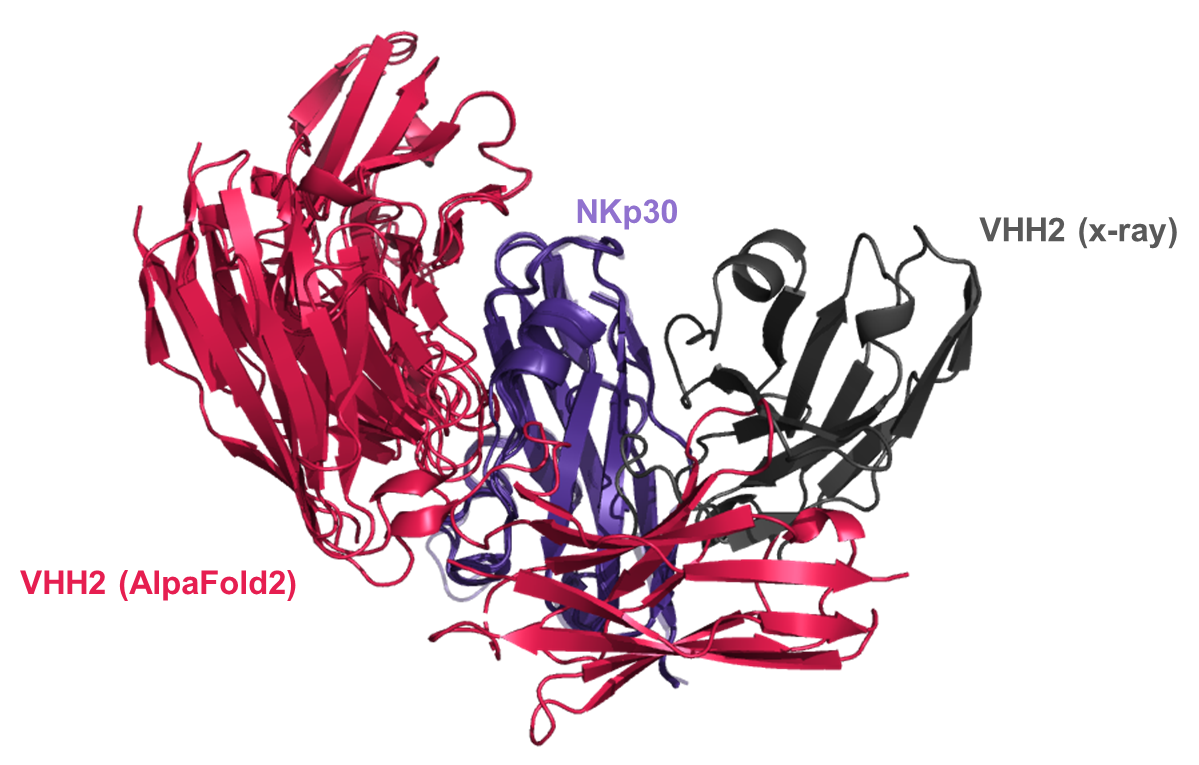


**Supplementary Figure 2**. Structural alignment of VHH2-NKp30 x-ray complex (PDB code 9FWW) and five AlphaFold2 generated VHH2-NKp30 complexes, superimposed on NKp30. AlphaFold2 derived VHH2 binding modes are shown in red and the experimental VHH2 binding mode is shown in gray.


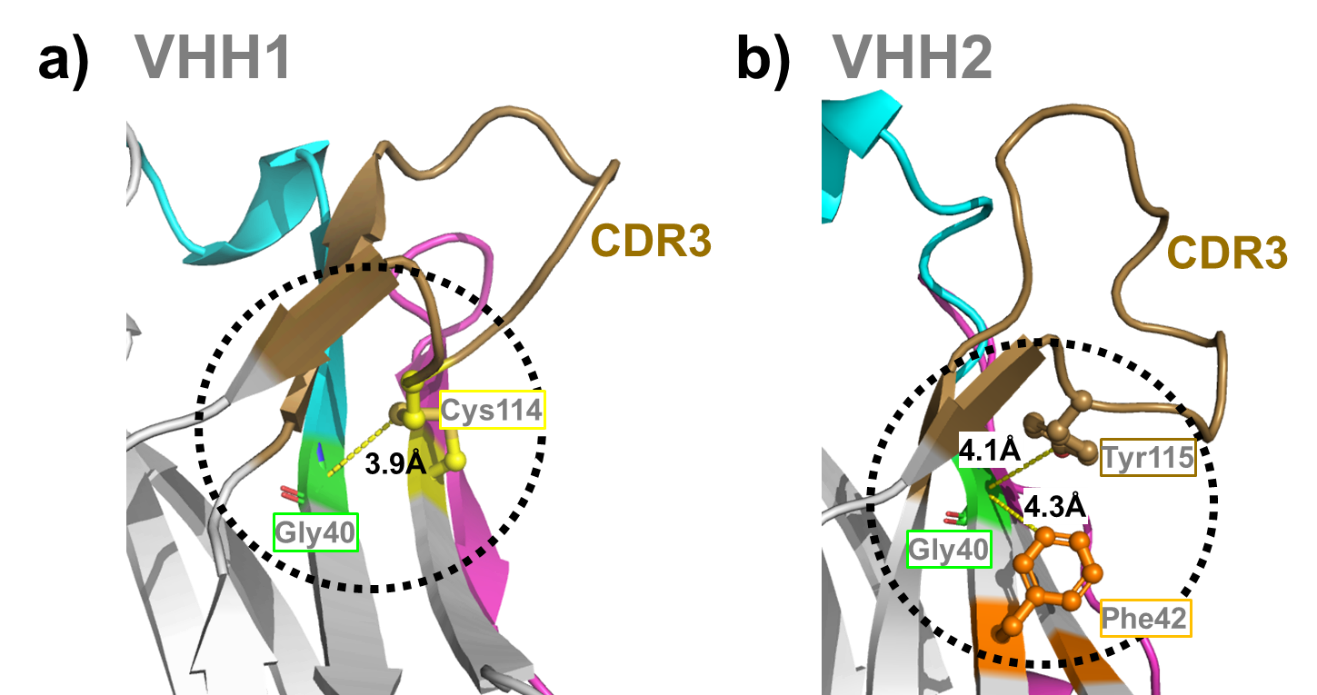


**Supplementary Figure 3**. Distance measurements of the Gly40 Cα atoms of a) VHH1 (PDB code 9FXF) and b) VHH2 (PDB code 9FWW) to most nearby residues (Cys114 in VHH1 and Phe42 & Tyr115 in VHH2) suggests that for both VHHs, the humanization variant G40S would influence the CDR3 conformation that might lead to a reduction of binding affinity.


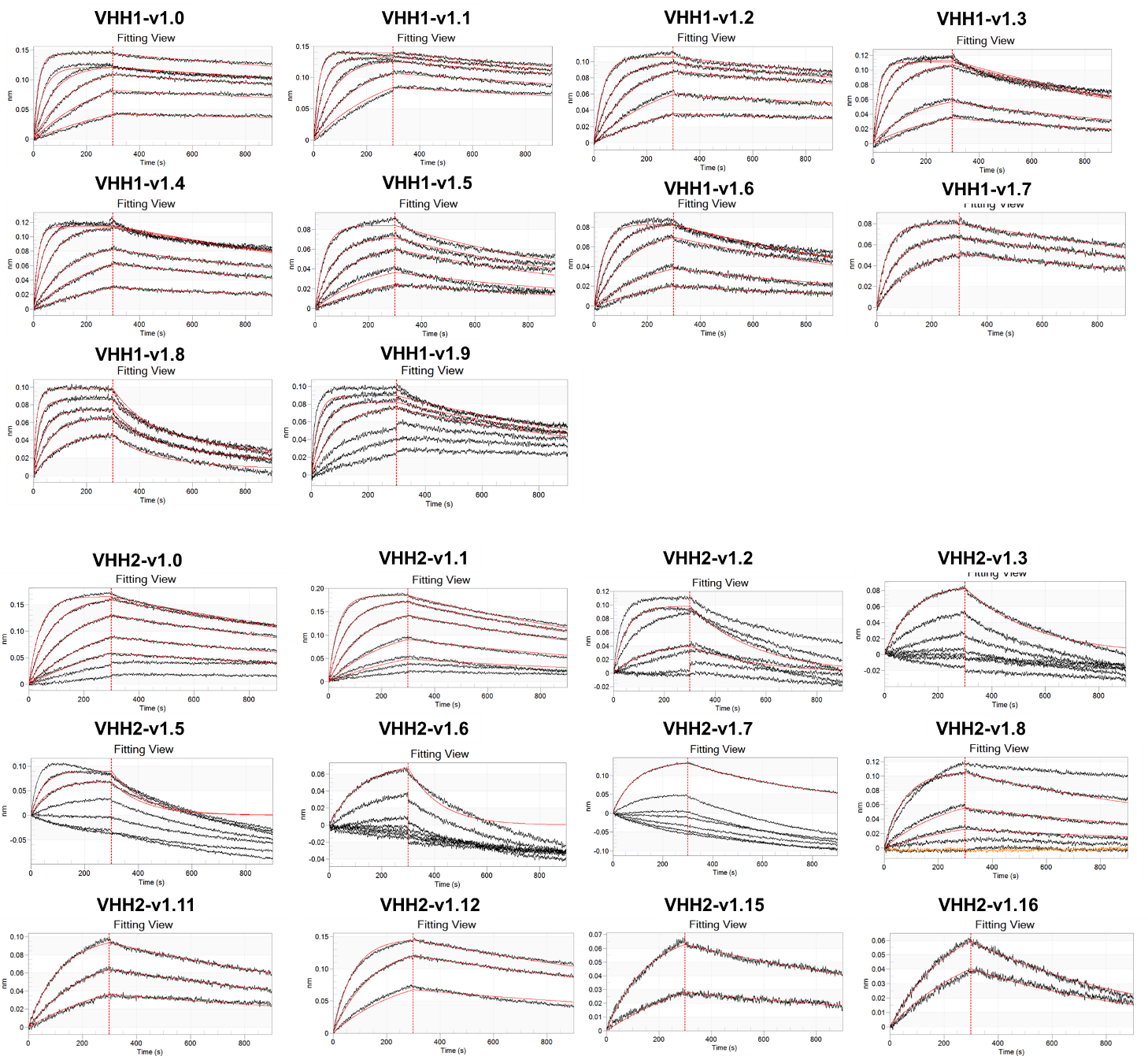


**Supplementary Figure 4**. BLI traces of VHH1 and VHH2 variants for which binding was observed.


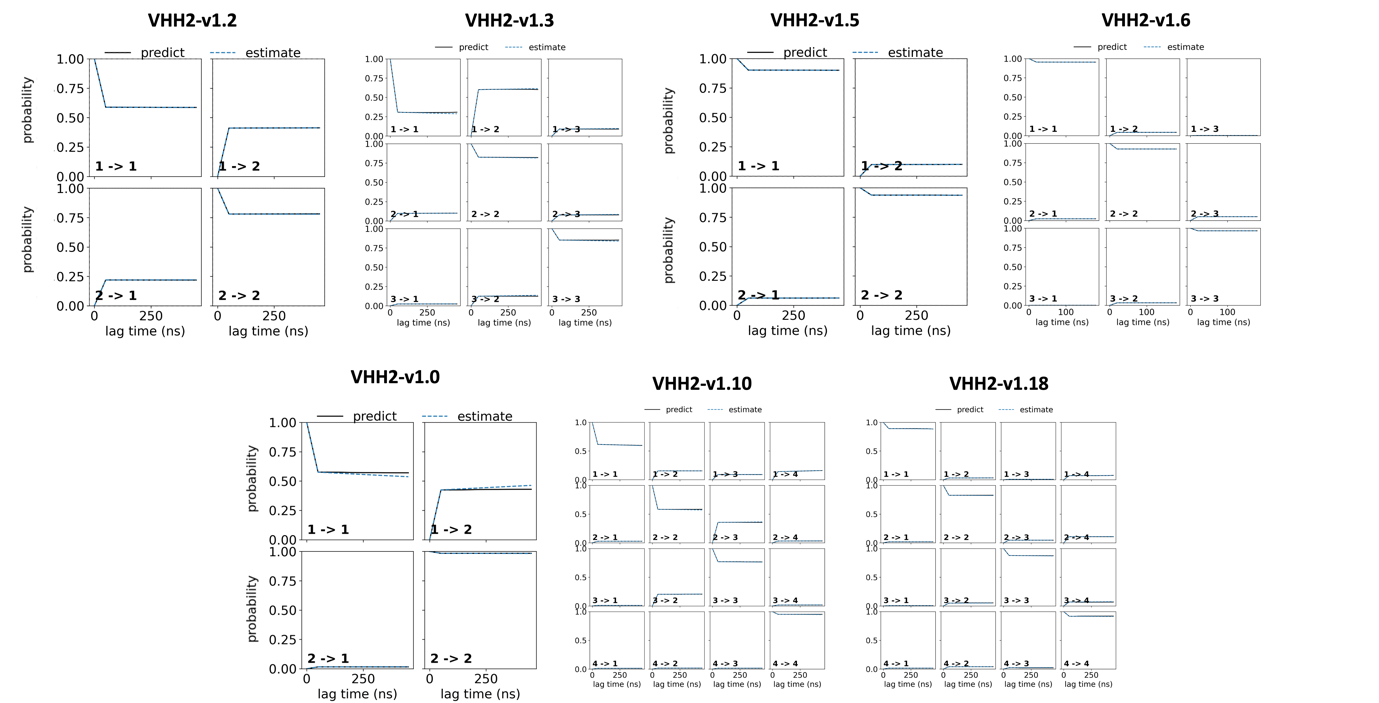


**Supplementary Figure 5**. Chapman-Kolmogorov tests to ensure reliability for the Markov-state models shown in Figure 6 of variants VHH2-v1.2, VHH2-v1.3, VHH2-v1.5, VHH2-v1.6, VHH2-1.0, VHH2-1.10, VHH2-1.18.
